# Graphene nanoribbons initiated from molecularly derived seeds

**DOI:** 10.1038/s41467-022-30563-6

**Published:** 2022-05-30

**Authors:** Austin J. Way, Robert M. Jacobberger, Nathan P. Guisinger, Vivek Saraswat, Xiaoqi Zheng, Anjali Suresh, Jonathan H. Dwyer, Padma Gopalan, Michael S. Arnold

**Affiliations:** 1 Department of Materials Science and Engineering, University of Wisconsin-Madison, Madison, WI 53706 USA; 2 Argonne National Laboratory, Center for Nanoscale Materials, Argonne, IL 60439 USA; 3 Department of Chemical and Biological Engineering, University of Wisconsin-Madison, Madison, WI 53706 USA

**Keywords:** Synthesis of graphene, Electronic properties and devices, Synthesis and processing

## Abstract

Semiconducting graphene nanoribbons are promising materials for nanoelectronics but are held back by synthesis challenges. Here we report that molecular-scale carbon seeds can be exploited to initiate the chemical vapor deposition (CVD) synthesis of graphene to generate one-dimensional graphene nanoribbons narrower than 5 nm when coupled with growth phenomena that selectively extend seeds along a single direction. This concept is demonstrated by subliming graphene-like polycyclic aromatic hydrocarbon molecules onto a Ge(001) catalyst surface and then anisotropically evolving size-controlled nanoribbons from the seeds along $\left\langle 110\right\rangle$ of Ge(001) via CH_4_ CVD. Armchair nanoribbons with mean normalized standard deviation as small as 11% (3 times smaller than nanoribbons nucleated without seeds), aspect ratio as large as 30, and width as narrow as 2.6 nm (tunable via CH_4_ exposure time) are realized. Two populations of nanoribbons are compared in field-effect transistors (FETs), with off-current differing by 150 times because of the nanoribbons’ different widths.

## Abstract (web summary)

The authors report on harnessing sp2-organic molecules as a basis for uniformly initiating the homoepitaxial-like, size-controlled, synthesis of sub-5 nm semiconducting graphene nanoribbons for electronics via chemical vapor deposition.

## Introduction

Semiconducting graphene nanoribbons are promising candidates for succeeding and/or complementing Si in logic microprocessors and III–V compounds in radio frequency devices^1–5^ and for integrating into emerging thin film^6^, optoelectronic^7^, spintronic^8,9^, and quantum devices^10,11^ because of their large current-carrying capacity^12^, high carrier velocity^13^, bandgap tunability, and outstanding thin-body electrostatic control^14^. To meet the demands of most of these applications, nanoribbons narrower than 5 nm are needed as they can have technologically relevant bandgaps arising from quantum confinement effects. The fabrication of nanoribbons this narrow is challenging and has been pursued via lithography and etching^15,16^, chemical/mechanical exfoliation of graphite^17^, unzipping carbon nanotubes^18,19^, synthesis templated within hexagonal boron nitride trenches^20,21^ or at SiC step edges^22–24^, and on-surface or solution-phase polymerization^25–28^. While many of these avenues are promising, numerous challenges remain including precise control over width, length, orientation, registration, and edge termination; periodic array formation; device integration; and translation to technologically relevant substrates. Substantial fundamental discovery of new synthetic strategies is needed before it will be possible to exploit nanoribbons in technology.

One promising strategy is to grow nanoribbons by chemical vapor deposition (CVD) on surfaces that promote highly anisotropic growth kinetics. Recent theoretical work has illuminated how graphene edge–substrate interactions can drive dramatic differences in the growth of graphene by CVD along different crystallographic directions of catalyst surfaces^29^. We recently exploited this phenomenon to directly grow armchair graphene nanoribbons from CH_4_ on technologically relevant Ge(001)^30^, Ge(001) epilayer on Si(001)^31^, and vicinal Ge(001) wafer platforms^32^. The anisotropic graphene evolution desirably results in nanoribbons self-aligned along Ge$\left\langle 110\right\rangle$ and with faceted armchair edges. The width of the nanoribbons increases slowly with time, for example at a rate of only 3 nm h^−1^ or smaller, opening the possibility for precisely controlling width by tailoring the duration of growth during CVD. The length of the nanoribbons increases many times faster than the width, enabling the synthesis of nanoribbons with aspect ratio that can exceed 30.

A major challenge, however, has been nanoribbon-to-nanoribbon width variation. The typical mean-normalized width polydispersity (standard deviation/mean, *σ/µ*) is 30–50%. This polydispersity arises in large part because the nanoribbons do not nucleate simultaneously or uniformly^30,33,34^. One possible solution to this challenge is to employ seeds to initiate the synthesis of all nanoribbons at the same time from a more consistent nucleus. Seeds fabricated lithographically by etching graphene have been used towards this end^34–36^. However, to synthesize sub-5 nm nanoribbons, sub-5 nm seeds are needed, which is a length scale difficult to access by contemporary lithography. Past attempts in the sub-5 nm regime have been hampered by substantial seed to seed variation, for example yielding populations of nanoribbons with an average width of 10 nm but a width polydispersity of more than 50%^35^. A more uniform nanoscale seed is therefore needed to progress towards the goal of monodisperse graphene nanoribbons via anisotropic CVD.

Here, we show that depositing polyaromatic hydrocarbon (PAH) molecules on a catalyst surface prior to nanoribbon synthesis can initiate and seed the subsequent growth of nanoribbons that can be tuned to be as narrow as 2.6 nm and that are substantially more uniform in width than nanoribbons grown without seeds. In the past, seeds and other seed-like features have provided a powerful avenue for initiating and manipulating the growth of two-dimensional materials, and have been leveraged to set lattice orientation^37^, control location^38–40^, create heterostructures^41^, and control grain boundary density and location^42,43^. Our study is distinct in that it demonstrates that, when coupled with directional growth behavior, seeds can be utilized to generate one-dimensional nanostructure analogs of two-dimensional materials, with widths <5 nm. It should also be noted that PAHs have been previously used as a carbon precursor (e.g., as a substitute for CH_4_) to grow continuous sheets of graphene^44–50^; and, moreover, PAHs have been fused inside carbon nanotubes^51–53^ and in films^54^ to create graphene nanoribbons. Our study is distinct in that it utilizes PAHs only as a means for uniformly initiating synthesis, and not as a chemical precursor. After sublimation on the catalyst surface, PAH-derived seeds evolve anisotropically via CH_4_ CVD, yielding nanoribbons that are isolated on surfaces for electronics, with controlled and tunable width.

## Results

**Fig. 1:**
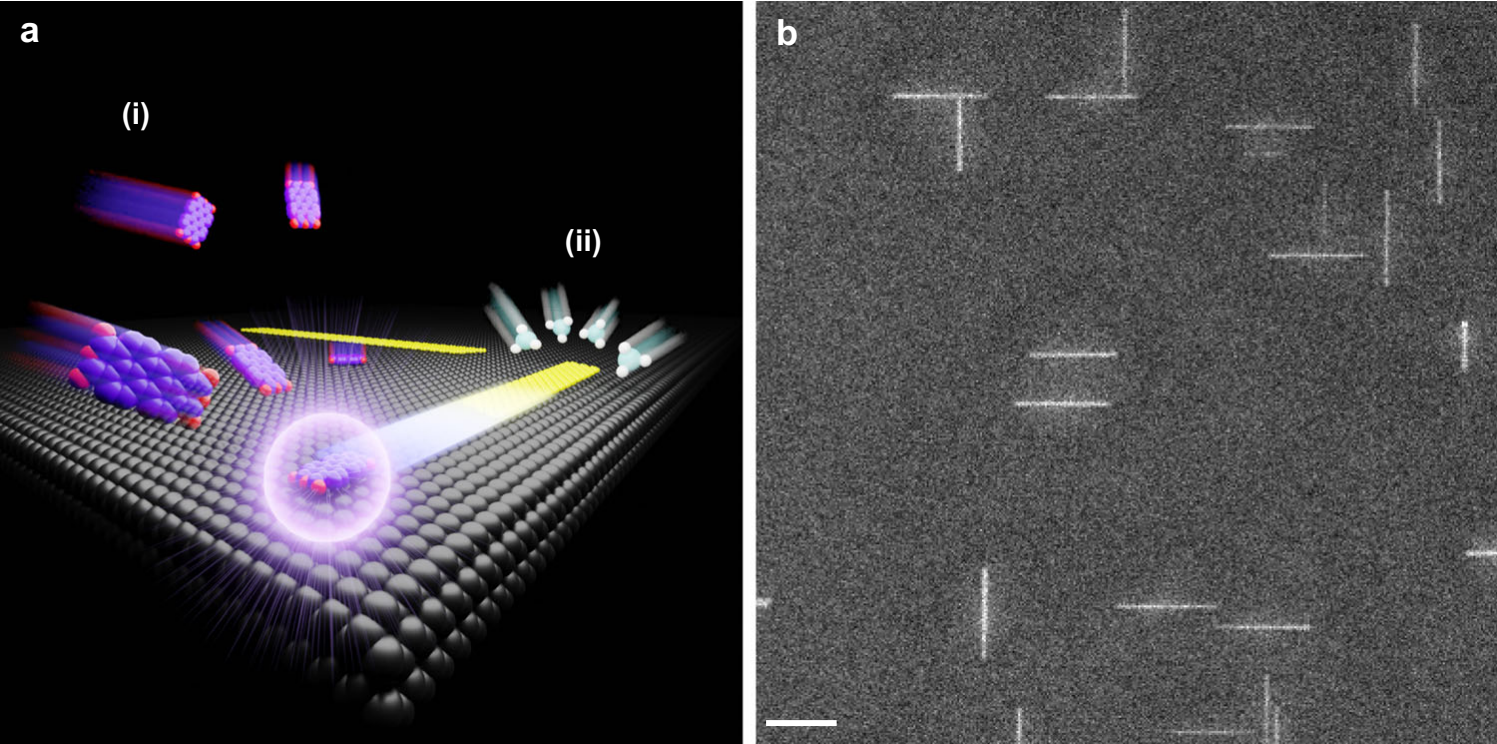
Nanoribbon synthesis from molecularly derived seeds. **a** Schematic diagram of the two main stages. (i) PAH molecules (red = oxygen and purple = carbon) are deposited at relatively low temperature onto Ge(001) (gray) from the vapor phase to form seeds. (ii) CH_4_ (light blue = carbon and white = hydrogen) is used to drive anisotropic growth via CVD and extend the seeds selectively along one direction, at high temperature, to yield narrow, armchair graphene nanoribbons. **b** Representative SEM image of nanoribbons initiated from PTCDA-derived seeds after 173 min of CH_4_ exposure. Scale bar is 200 nm.

Our strategy for synthesizing nanoribbons from PAH-derived seeds is illustrated in Fig. 1a and comprised of two stages: (i) initiation and (ii) anisotropic growth. In (i), PAHs are sublimed onto Ge(001) at relatively low temperature to initiate the subsequent synthesis of nanoribbons during stage (ii). Perylenetetracarboxylic dianhydride (PTCDA) and pentacene are employed as model PAHs, here, because of their planarity and relatively low sublimation temperature. In stage (ii), CH_4_ is flowed through the CVD reactor at high temperature to anisotropically evolve nanoribbons from PAH-derived seeds. The detailed protocols used to implement stages (i) and (ii), in practice, are schematically depicted in Supplementary Fig. 1. Examples of 12 nm wide nanoribbons initiated from PTCDA-derived seeds are shown in Fig. 1b after 173 min of CH_4_ exposure at growth temperature. These molecularly seeded nanoribbons self-orient along Ge$\left\langle 110\right\rangle$ and have a large aspect ratio (e.g., 28 in Fig. 1b), similar to nanoribbons that spontaneously grow on Ge(001) without seeds. A fundamental difference, however, is that the molecularly seeded nanoribbons are substantially more uniform in width (as quantified in detail, below). Once initiated, graphene islands grow with a large aspect ratio during stage (ii) because of the natural propensity of graphene islands to facet with armchair edges on Ge(001)^34^, self-rotate^36^, and grow highly anisotropically, in which edges parallel (unaligned) with Ge$\left\langle 110\right\rangle$ grow slowly (quickly)^34^, likely as a result of kinetic barriers for edge attachment that are modulated by edge-Ge bonding configuration^32,55^.

Two conditions that are critical to initiating the synthesis of nanoribbons from PAH-derived seeds are (1) implementing the growth stage in a regime in which spontaneous nucleation is suppressed but anisotropic graphene evolution from existing seeds and domains is promoted, and (2) controlling PAH dose. To meet these conditions, the general procedure is as follows. First, the Ge substrates are annealed in 33% H_2_ balanced by Ar at 920 °C to remove carbon-based contaminants prior to PAH deposition. Removing these contaminants suppresses spontaneous nucleation, avoiding the unwanted initiation of nanoribbons by CH_4_ alone during the later growth stage. Second, PAHs are deposited by cooling the Ge substrates to 165 °C (for PTCDA) or 125 °C (for pentacene) and subliming the PAHs onto the Ge from an upstream quartz crucible containing PAH powder at 220 °C (for PTCDA) or 160 °C (for pentacene). The dose can be generally controlled via crucible temperature, which affects the vapor pressure of the PAH, and/or sublimation time. After dosing the Ge with PAHs, the reactor is cooled to room temperature, CH_4_ is introduced into the gas stream, and the temperature is rapidly increased (over 5 min) to 920 °C to promote graphene nanoribbon evolution. On annealed Ge(001) surfaces, a window of growth conditions exists at CH_4_ concentrations between 0.5% and 0.9% (at an H_2_ concentration of 33%) in which nanoribbon growth can be driven from seeds without appreciable spontaneous nucleation. In the data below, a CH_4_ concentration of 0.56% is utilized, intentionally chosen near the bottom of this range to exploit the fact that growth rate anisotropy increases with decreasing CH_4_ concentration on Ge(001), thereby yielding nanoribbons with high-aspect ratio^30–32^.

Control experiments adopting this protocol, but eliminating the deposition of PAHs, fail to appreciably yield nanoribbons (Supplementary Fig. 2), confirming that nanoribbons are unable to spontaneously nucleate from the supply of CH_4_, alone, in the absence of PAH-derived seeds. Likewise, control experiments adopting this protocol, but forgoing the delivery of CH_4_ to the Ge substrate, show that PAHs themselves are unable to form nanoribbons (Supplementary Fig. 2). These two experiments verify that seed formation during the initiation stage and nanoribbon synthesis during the anisotropic growth stage are distinct processes that can be independently controlled. Supplementary Fig. 3 confirms that nanoribbon density increases with increasing PAH sublimation dose during the initiation stage until the density becomes so high that nanoribbons begin to grow into each other, beyond which higher doses of PAHs template the synthesis of disordered carbon films rather than graphene nanoribbons. It is worth noting that, unlike nanoribbon density, the length of the nanoribbons does not increase with PAH dose, further confirming that the PAH dose initiates nanoribbons but that PAHs themselves are unable to drive the growth of nanoribbons. Rather, the growth, length, and width of the nanoribbons are independently controlled by the CH_4_ exposure time (as shown in further characterization below). It is also worth noting that, without the introduction of PAHs, a short burst of CH_4_ (above the CH_4_ concentration needed to induce spontaneous nucleation) primarily initiates the synthesis of polydisperse, low-aspect ratio graphene crystals (Supplementary Fig. 4). These low-aspect ratio features rapidly evolve during the burst, likely because of the super-linear dependence of growth rate on CH_4_ partial pressure^30–32^, as opposed to forming homogenous sub-2 nm graphene seed crystals capable of initiating the uniform synthesis of nanoribbons. This observation indicates that CH_4_, alone, is incapable of generating nanoribbons with the uniformity observed, here, even further confirming the role of the PAH dose in uniformly initiating nanoribbon synthesis.

**Fig. 2:**
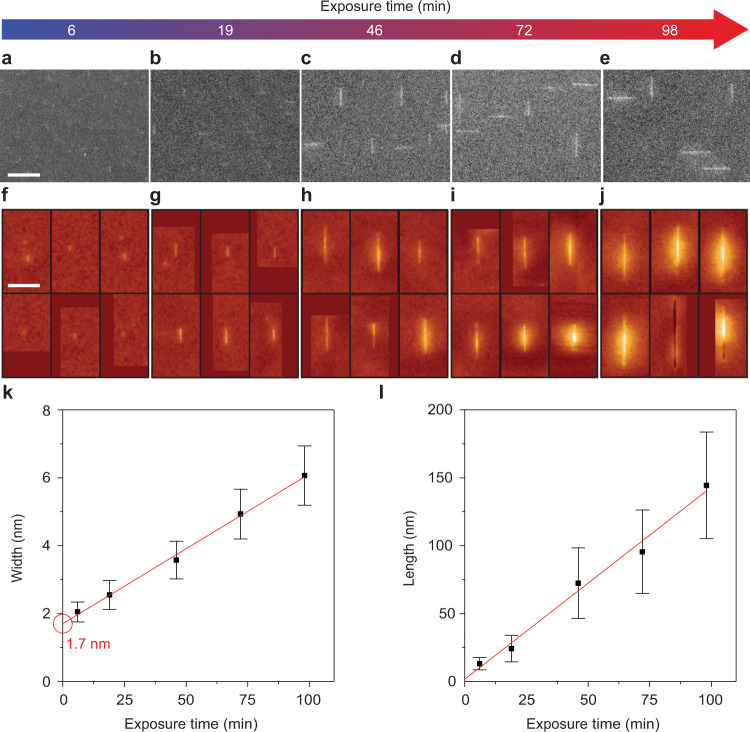
Anisotropic evolution from PTCDA-derived seeds. **a**–**e** SEM images of nanoribbons after CH_4_ exposure times of 6, 19, 46, 72, and 98 min. Scale bar is 200 nm. **f**–**j** STM images of nanoribbons after exposure times of 6, 19, 46, 72, and 98 min (applied bias = 2 V, tunneling current = 0.1 nA). Scale bar is 100 nm. Color is scaled to topographic height, with dark red being lowest and light yellow being highest. **k**, **l** Plots of width and length versus exposure time. Red lines are linear best fits. Red circle indicates where the linear fit intercepts the *y*-axis and defines the effective seed size of 1.7 nm. Error bars are standard deviation of width (**k**) and length (**l**).

Figure 2 evidences that the width and length of the nanoribbons can be intentionally controlled by varying CH_4_ exposure time, that nanoribbons effectively initiate growth from seeds that are only 1.7 nm in diameter, and that nanoribbons with aspect ratio greater than 10 and width as narrow as 2.6 nm can be realized. Figure 2a–e show scanning electron microscopy (SEM) images of nanoribbons initiated from PTCDA-derived seeds after various CH_4_ exposure times. The nanoribbons are too small to clearly resolve via SEM after 6 and 19 min of exposure in Fig. 2a, b but are more easily imaged after 46, 72, and 98 min of exposure in Fig. 2c–e. The nanoribbons become wider and longer with increasing CH_4_ exposure time. An additional SEM image after 203 min of exposure is provided in Supplementary Fig. 5 showing continued evolution with longer exposure times.

Scanning tunneling microscopy (STM) is used to image the nanoribbons with higher resolution and quantify their width and length (see representative images in Fig. 2f–j with additional large-area scans in Supplementary Fig. 6). The width linearly increases with CH_4_ exposure time and is 2.1 ± 0.3, 2.6 ± 0.4, 3.6 ± 0.6, 4.9 ± 0.7, and 6.1 ± 0.9 nm after 6, 19, 46, 72, and 98 min of exposure, respectively (Fig. 2k, with width histogram distributions in Supplementary Fig. 7). One goal of molecular scale seeding is to gain control over nanoribbon width by tailoring CH_4_ concentration or exposure time. Here, the change in width with respect to time is remarkably slow, only 2.6 nm h^−1^, enabling precise control over nanoribbon width by tailoring CH_4_ exposure time.

**Fig. 3:**
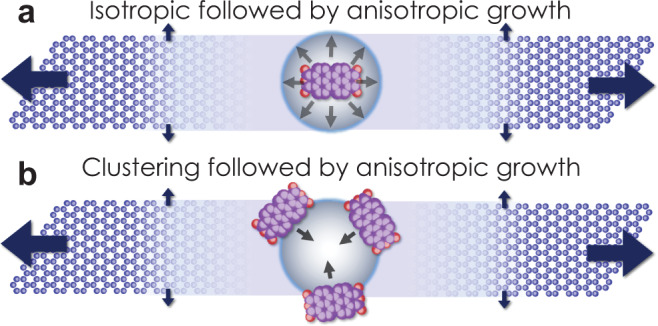
Schematic depiction of two possible nanoribbon initiation pathways from PAH-derived seeds. Here, graphene is in blue and PTCDA molecule is in purple (carbon) and red (oxygen). **a** Individual PAH molecules or fragments of PAH molecules grow relatively isotropically from CH_4_ to a critical size before anisotropic nanoribbon evolution begins. **b** Multiple PAH molecules or fragments of PAH molecules cluster to form seeds that are effectively larger in size (e.g., 1.7 nm for PTCDA, based on Fig. 2 data) that then initiate the anisotropic growth of nanoribbons.

Extrapolating a best-fit line to an exposure time of 0 min yields an estimate of 1.7 nm for the effective seed size prior to CH_4_ exposure. The meaning behind this initial size is not yet clear. One possibility is that individual PAH molecules or fragments of PAH molecules grow relatively isotropically to an effective seed size of 1.7 nm before anisotropic growth begins (as depicted in Fig. 3a). Another possibility is that multiple PAH molecules or fragments of PAH molecules cluster to form seeds that are effectively 1.7 nm in diameter that then template the anisotropic growth of nanoribbons (as depicted in Fig. 3b). Previous analysis of the stability of PAHs on Cu(111), which like Ge is a weakly interacting catalyst surface, determine that PAHs dehydrogenate at temperatures >500 °C, are stable against decomposition into smaller fragments at temperatures up to 1000 °C, and can covalently dimerize and form small clusters^46^—providing a pathway towards the incorporation of non-fragmented PAHs either as individual molecules or fused clusters in either of the scenarios presented in Fig. 3. Prior studies have moreover shown that PTCDA molecules, prior to their dehydrogenation, are mobile on Ge(001) at temperatures >180 °C and that non-covalent interactions among PTCDA molecules are favorable under particular arrangements leading to clustering^56,57^. While their mobility following dehydrogenation has not yet been studied, the PAH molecules studied, here, likely remain hydrogenated throughout the initiation stage and at the beginning of the nanoribbon growth stage (as the temperature is increased from room temperature to 920 °C) thereby providing the opportunity for PAH diffusion and/or clustering and further establishing the plausibility of the scenario presented in Fig. 3b (but not excluding the scenario in Fig. 3a). Imaging of PTCDA molecules deposited on Ge(001) in situ in a STM preparation chamber, before and after a high-vacuum ~900 °C flash, evidence that molecular species survive and features <2.5 nm in size form at high temperature (Supplementary Fig. 8). Although the density of PTCDA adsorbates and the high vacuum annealing conditions in these in situ experiments differ from the conditions used during actual nanoribbon synthesis in the CVD chamber, these data support the hypotheses outlined in Fig. 3. Future experiments will be needed to better elucidate the seed-to-nanoribbon transition.

Interestingly, a bimodal length distribution is consistently observed, indicating two modes of growth along the nanoribbon long axis. Considering both modes, the nanoribbon length increases linearly with exposure time and is 13 ± 4.5, 24 ± 9.7, 72 ± 26, 95 ± 31, and 144 ± 39 nm after 6, 19, 46, 72, and 98 min of exposure, respectively (Fig. 2l). This bimodal distribution is most clearly resolved after longer exposure times (Supplementary Fig. 9). A minority of the nanoribbons (about 30%) are about one-half the length of the others, indicating that the majority population grows bidirectionally whereas the minority population grows unidirectionally away from seeds (Supplementary Figs. 9, 10). The bidirectional growth of nanoribbons has been observed previously from larger, lithographically patterned seeds^34,35^. The length of the bidirectional population of nanoribbons increases at a rate of 98 nm h^−1^. The aspect ratio of the bidirectional nanoribbons increases with increasing exposure time, exceeding 12 at a width of 2.6 nm after 19 min and reaching 33 at a width of 8.5 nm after 173 min (Supplementary Fig. 11). The increase in aspect ratio with time occurs because the seeds begin as low-aspect ratio structures with non-zero widths. The nanoribbons then evolve anisotropically from the seeds with a growth rate in the length direction that is 32 times the growth rate in the width direction (for the case of bidirectional nanoribbons). It is not yet clear why the minority population of nanoribbons grows only along one direction. One possibility is that the growth is obstructed along one direction. Towards this end, a topographical feature is present at one end of some minority population nanoribbons, although this feature is not consistently observed (Supplementary Fig. 10).

**Fig. 4:**
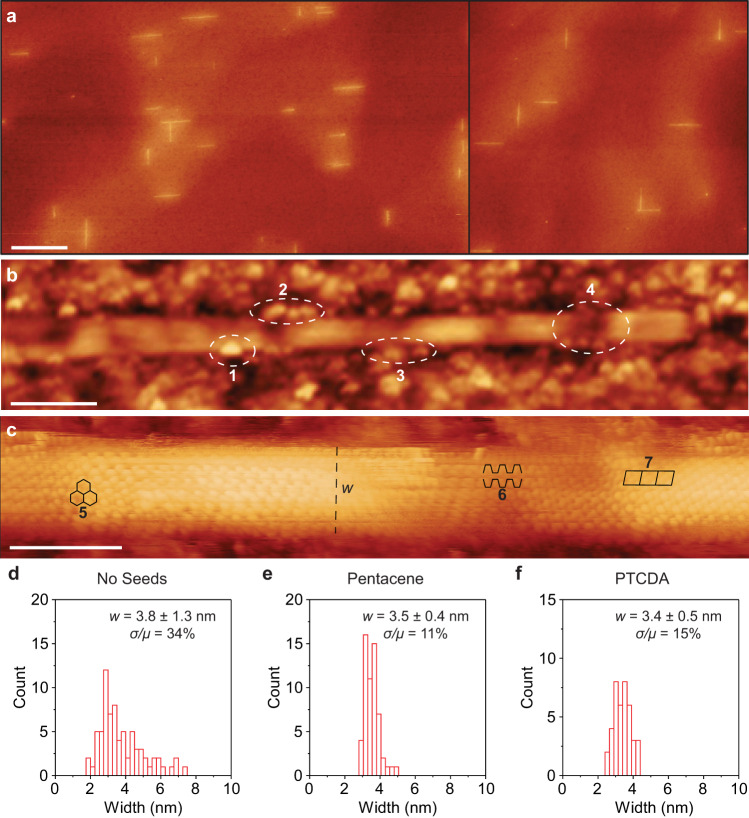
Width distribution of PTCDA-derived seeds. **a** Large area STM images of nanoribbons initiated from PTCDA-derived seeds after 46 min of CH_4_ exposure (applied bias = 2 V, tunneling current = 0.1 nA). Scale bar is 200 nm. **b** High magnification STM image of an individual nanoribbon (applied bias = 2 V, tunneling current = 0.1 nA). Scale bar is 10 nm. Features 1–3 mark GeO_*x*_ species and/or adsorbates on the bare Ge surface encroaching upon or directly touching the nanoribbon edges (resulting from exposure of the substrate to ambient air during transfer from the CVD reactor to the STM chamber). Feature 4 highlights topographical variation in the Ge underneath the nanoribbons (resembling the shallow hills and valleys that become more prominent in wider nanoribbons because of the nanofaceting of the Ge(001) surface under graphene during synthesis). **c** High magnification STM image of an individual nanoribbon (applied bias = 0.2 V, tunneling current = 1 nA). Scale bar is 3 nm. Features 5–7 mark honeycomb, intervalley scattering with periodicity of *λ*_f_ (wavelength of electrons near the high symmetry K-point of the first Brillouin zone of graphene), and $\sqrt 3$ quasiparticle interference patterns, respectively, in the nanoribbon interior. In some instances, these patterns change from scan to scan, likely concurrent with apparent changes in GeO_*x*_ species and/or adsorbates near the edges or tip (Supplementary Fig. 13). *w* highlights the nanoribbon width. An additional nanoribbon is characterized by high magnification STM in Supplementary Fig. 14. **d**–**f** Width histograms of nanoribbons initiated without seeds, with pentacene-derived seeds, and with PTCDA-derived seeds after 46 min of CH_4_ exposure. Mean (*µ*), standard deviation (*σ*), and polydispersity (*σ*/*µ*) are specified, where *w* = *µ* ± *σ*. STM images in **a**–**c** are scaled to topographic height, with dark red being lowest and light yellow being highest.

Figure 4 quantifies the improvement in width polydispersity that results from initiating nanoribbon growth from PAH-derived seeds. Here, unseeded nanoribbons initiated by spontaneous nucleation are compared to nanoribbons initiated from pentacene- and PTCDA-derived seeds—all after 46 min of evolution from CH_4_. The unseeded nanoribbon substrates are prepared by reducing the duration of the pre-synthesis 920 °C anneal in 33% H_2_ from 90 to 15 min, to enable spontaneous nucleation, and then forgoing PAH deposition. STM scans with a pixel size ranging from 0.1 to 0.2 nm are collected to characterize the width of 74, 40, and 58 different nanoribbons from unseeded, PTCDA-initiated, and pentacene-initiated samples, respectively. Large-area scans are shown in Fig. 4a; higher magnification scans are presented in Fig. 4b, c; and, histograms of the width distributions are shown in Fig. 4d–f. The unseeded nanoribbons have a width of 3.8 ± 1.3 nm (corresponding to a mean normalized standard deviation of 1.3 nm/3.8 nm = 34%). In contrast, the widths of the pentacene- and PTCDA-initiated nanoribbons are 3.5 ± 0.4 and 3.4 ± 0.5 nm, corresponding to mean-normalized standard deviations of 11% and 15%, respectively. Thus, the width polydispersity of molecularly seeded nanoribbons is substantially better than nanoribbons that spontaneously nucleate without seeds. Without seeds, nucleation is likely driven by carbon-based contaminants (that would otherwise be removed with additional H_2_ annealing), and the resulting nanoribbon polydispersity can therefore be attributed to non-uniformities associated with these contaminants in addition to variation in nucleation time. In contrast, the PAH-derived seeds more simultaneously and uniformly initiate the synthesis of nanoribbons. Like the width polydispersity, the length polydispersity of the bidirectional population of nanoribbons is roughly 10% (Supplementary Fig. 9). Histograms of the length and aspect ratio of the nanoribbons analyzed in Fig. 4 are provided in Supplementary Fig. 12.

The observation of honeycomb, intervalley scattering with period of 0.35 nm (near the wavelength of electrons at the high-symmetry K-point of the first Brillouin of graphene, *λ*_f_, of 0.37 nm), and $\sqrt 3$ quasiparticle interference patterns in the nanoribbon interiors (Fig. 4c) confirms that the interiors of the nanoribbons are highly crystalline and demonstrates that electronic states are delocalized across the nanoribbon width. The observation of these patterns moreover confirms the armchair orientation of the nanoribbons^58–62^. Additional high-resolution STM data are shown in Supplementary Figs. 13 and 14. Height maps (measured via atomic force microscopy) and Raman spectra are moreover shown in Supplementary Figs. 15 and 16, respectively.

**Fig. 5:**
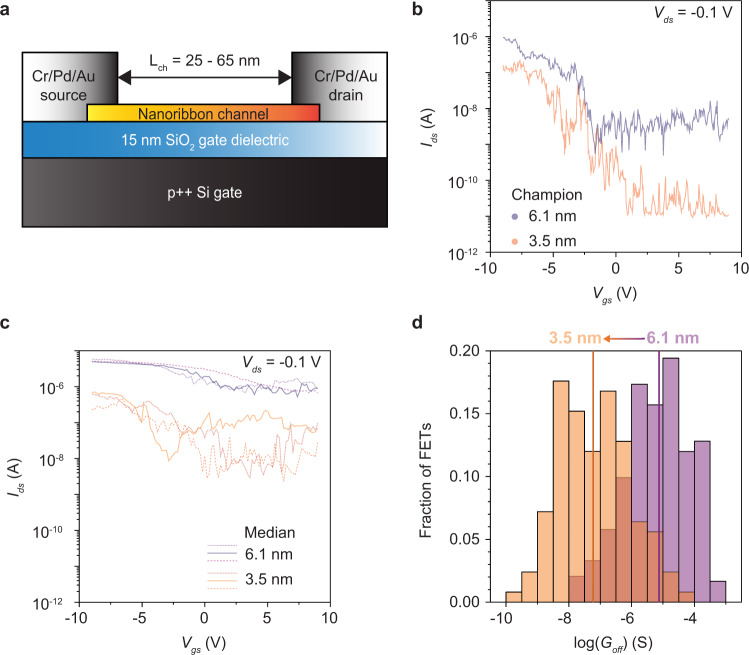
FET measurements of 6.1 and 3.5 nm wide, PTCDA-derived nanoribbons. **a** Nanoribbon FET architecture. **b** and **c** Source–drain current (*I*_ds_) versus source–gate bias (*V*_gs_) at a source–drain bias (*V*_ds_) of −0.1 V for one champion (**b**) and three median (**c**) FETs for each nanoribbon width population. **d** Histograms of off-state conductance (*G*_off_). The median off-state conductance improves by 2.2 orders of magnitude in the narrower ribbons. Purple and orange data correspond to nanoribbons with average widths of 6.1 and 3.5 nm, respectively.

Two different populations of nanoribbons (width = 3.5 nm, PTCDA-initiated, 46 min of CH_4_ exposure; and, width = 6.1 nm, PTCDA-initiated, 98 min of CH_4_ exposure) are integrated into field-effect transistors (FETs) in Fig. 5. The nanoribbons are transferred to degenerately doped Si wafers with 15 nm of thermally grown SiO_2_, which serves as a universal back-gate for the FETs. A sacrificial polymer film is used to transfer the nanoribbons from Ge to SiO_2_, in conjunction with etching of the Ge growth substrate. Representative SEM images of transferred nanoribbons and a FET are shown in Supplementary Fig. 17. After transfer, Cr/Pd/Au top-contacts with thickness of 0.7/9.3/10 nm are deposited by thermal evaporation to define a channel length of 25–65 nm (Fig. 5a). Source–drain current (*I*_ds_) versus source–gate bias (*V*_gs_) at a source–drain bias (*V*_ds_) of −0.1 V is compared for champion (Fig. 5b) and median FETs (Fig. 5c). The devices turn on at negative *V*_gs_, demonstrating p-type behavior, as expected for nanoribbon FETs with Pd contacts measured in ambient air at room temperature. The champion 3.5 nm nanoribbon FETs simultaneously exhibit high on/off ratio and high on-state conductance. For example, 13 FETs display an on/off ratio ranging from 1.1 × 10^3^ to 8.1 × 10^3^, in which the on-state conductance ranges from 1.6 to 6.5 μS, and the corresponding on-state conductance normalized by width ranges from 460 to 1800 μS μm^−1^. The on-state conductance normalized by width is similar to that reported for champion nanoribbon FETs in the literature produced via chemical/mechanical exfoliation of graphite^63^ and bottom-up organic synthesis^1^. Linear plots of *I*_ds_ versus *V*_gs_ and *I*_ds_ versus *V*_ds_ sweeps are presented in Supplementary Figs. 18, 19. More than 125 and 242 different 3.5 and 6.1 nm nanoribbon FETs, respectively, are measured, and histograms of off-state conductance are compared in Fig. 5d. The median off-conductance is suppressed by 2.2 orders of magnitude in the narrower ribbons, which are expected to exhibit smaller off-conductance because of their larger bandgaps^64^ and transport gaps^65^. Ultimately, arrays of unidirectionally aligned nanoribbons will be needed for high-performance FETs. Towards this goal, nanoribbons with a highly preferred orientation are obtained by initiating synthesis from PAH-derived seeds on vicinal Ge(001) substrates (Supplementary Fig. 20). Future comparison of the charge transport properties of nanoribbons grown from molecularly derived seeds on vicinal Ge(001) versus Ge(001) substrates will be needed; prior studies of unseeded nanoribbons on both surfaces have observed similar charge transport properties^32^.

## Discussion

These results exhibit the concept of synthesizing graphene nanoribbons via directional addition to molecular scale and molecularly derived seeds. The results specifically demonstrate that the sublimation of PAHs onto a catalyst surface generates seeds with molecular dimensions (here, as small as 1.7 nm) and that these seeds can be exploited to generate armchair graphene nanoribbons as narrow as 2.6 nm, when coupled with directional growth phenomena that selectively extend the seeds along Ge$\left\langle 110\right\rangle$ (here, by CH_4_). The two stages of nanoribbon synthesis—initiation and anisotropic growth—can be separately tailored, enabling control over both nanoribbon density and size. The nanoribbons slowly increase in width at a rate of only 2.6 nm h^−1^, enabling sensitive width control. The resulting width polydispersity is three times better than nanoribbons that spontaneously nucleate without seeds, with a mean-normalized standard deviation as tight as 11%, demonstrating the excellent uniformity of the PAH-derived seeds and the ability of the seeds to simultaneously initiate the growth of nanoribbons. These nanoribbons are long enough for modern-day high-performance field effect transistors, in which the channel length is only 10’s of nanometers.

The partial pressure of CH_4_ used in this synthesis is large enough to drive the anisotropic growth of graphene islands that have already nucleated but is not large enough to drive their nucleation (Supplementary Fig. 2). Alternatively, a short burst of higher CH_4_ partial pressure can be used to nucleate nanoribbons, but the nuclei of graphene that form rapidly expand because of the super-linear dependence of growth rate on CH_4_ partial pressure (Supplementary Fig. 4) and therefore undesirably initiate large and polydisperse nanoribbons of uncontrolled size. The separation of nanoribbon initiation and growth into separate processes overcomes this problem. The dosing of the Ge(001) surface with PAH molecules seeds the formation of graphene islands that can then grow and evolve anisotropically via CH_4_ exposure. The control experiments presented in Supplementary Fig. 2 demonstrate that the PAH dose itself is insufficient to drive growth, and increasing the PAH dose increases the density of nanoribbons rather than altering their size or shape (Supplementary Fig. 3), further evidencing the role of the PAH in seeding but not driving growth. The data in Fig. 2k show that the effective seed size is only 1.7 nm, consistent with one to a few PAH molecules or molecule fragments. Determining the exact seed structure will require future studies; although, STM characterization of seed molecules before and after a 900 °C flash establish that molecular species and molecular-scale carbon features survive at high temperature. Regardless, the data clearly show that dosing the Ge(001) surface with PAHs initiates the synthesis of highly uniform nanoribbons.

Results from previous studies explain why graphene nuclei and seeds grow anisotropically into nanoribbon-shaped islands rather than more isotropic structures (e.g., hexagons) on Ge(001). Way et al. specifically examined growth from the edges of circular graphene islands transferred onto Ge(001) at different orientations with respect to the Ge lattice, finding that the growing islands always become armchair edge faceted and that the growth rate of island edges tends towards zero for edges parallel to Ge$\left\langle 110\right\rangle$. The edge growth rate increases as the angle between the edge and the nearest Ge$\left\langle 110\right\rangle$ orientation increases^34^. As a result of this dependence, any graphene island on Ge(001) with an armchair lattice orientation parallel to Ge$\left\langle 110\right\rangle$ will grow highly anisotropically. The results here show that this behavior persists for graphene islands with single nanometer dimensions. In general, the evolution of graphene islands into shapes that do not possess the six-fold symmetry of the graphene lattice has been explained by edge–substrate interactions that break this symmetry. Prior density functional theory calculations have indeed revealed strong armchair edge–Ge(001) binding along Ge$\left\langle 110\right\rangle$ surface directions^32^. Prior research on lithographically defined graphene seeds on Ge(001) has moreover shown that small seeds <10 nm in diameter automatically self-rotate on Ge(001) until the armchair lattice orientation of the seed is parallel to Ge$\left\langle 110\right\rangle$^36^. This self-rotation establishes the relative epitaxial orientation needed for the subsequent anisotropic shape evolution of seeds into high aspect ratio islands, thereby enabling the consistent evolution of molecularly derived seeds into long and narrow nanoribbons.

Beyond the data presented here, future experiments will be needed to directly image the shape and size of PAH-derived seeds, elucidate how these aspects are affected by PAH composition (e.g., pentacene versus PTCDA versus other molecules), and analyze the early stages of seed-to-nanoribbon evolution. Additional work will also be needed to image the edge structure of the nanoribbons with atomic resolution and to understand how both the initiation and anisotropic growth stages can be tailored to ensure that the edges are smooth by exploiting the natural tendency of graphene crystal growth on Ge(001) to produce faceted, armchair edges^30,31,33^. Resolution of edge structure in the high magnification STM images presented in Fig. 4c and Supplementary Figs. 13 and 14 is challenged by edge–Ge bonding^32^, GeO_*x*_ species and adsorbates on the bare Ge surface encroaching upon or directly touching the edges (resulting from exposure of the substrate to ambient air during transfer from the CVD reactor to the STM chamber), and topographical variations in the underlying Ge (resembling the shallow hills and valleys that become more prominent in wider nanoribbons because of the nanofaceting of the Ge(001) surface under graphene during synthesis). Therefore, resolving the edges in the future may require either nanoribbon growth in situ within an STM chamber (to avoid contamination of the Ge surface via ambient exposure) or transfer of the nanoribbons from the Ge to a flatter, more passivated surface that is more conducive to atomic resolution STM following ambient exposure. It is worth noting that previous STM studies of wider nanoribbons on Ge(001) have identified long (10 nm) stretches of smooth, faceted edges^31^. Future experiments will also be needed to understand and separate the effects of Schottky barriers, insulating residues introduced by transfer, contact length, edge disorder, and substrate disorder on the charge transport properties of the nanoribbons. Increasing the nanoribbon length and aspect ratio may also be viable in the future by tailoring the precursor and catalyst substrate.

## Methods

### Preparation of the Ge(001) surface

Ge(001) (Wafer World, resistivity >40 Ω-cm) is cleaved along Ge$\left\langle 110\right\rangle$ directions into 1.0 × 0.75 cm^2^ rectangular pieces using a diamond scribe. The substrates are loaded into a horizontal quartz tube furnace with an inner diameter of 34 mm and three heated zones. Next, the tube is evacuated to ~10^−4^ torr for 10 min and then filled to atmospheric pressure via the flow of 200 sccm of Ar (purity of 99.999%) and 100 sccm of H_2_ (purity of 99.999%). The substrates and all three zones of the furnace are heated to 920 °C under these flow conditions for 1.5 h (Supplementary Fig. 1a). This duration is sufficient to suppress the spontaneous nucleation of nanoribbons during subsequent growth stages.

### In-situ deposition of small molecules

After annealing, the furnace and substrates are cooled to approximately room temperature (Supplementary Fig. 1b). The substrates are heated to 165 °C (for PTCDA) or 125 °C (for pentacene) in the downstream zone of the furnace in an environment of 200 sccm of Ar and 100 sccm of H_2_ (Supplementary Fig. 1c, d). Under the same environment, a quartz boat filled with a powder of either PTCDA (Luminescence Technology, LT-S920, sublimed grade >99%) or pentacene (Sigma-Aldrich, 684848, sublimed grade >99.9%) is inserted into the upstream zone of the furnace at 220 °C (for PTCDA) or 160 °C (for pentacene) without breaking the system’s seal—to begin sublimation of the molecules (Supplementary Fig. 1e). The dose of the molecule is varied by controlling the duration of sublimation (typically 20–30 min for the syntheses presented in Figs. 1–5) before the PAH source material and substrates are rapidly cooled to room temperature (Supplementary Fig. 1f).

### Anisotropic graphene nanoribbon growth via chemical vapor deposition of CH_4_

After sublimation of the small molecules, a flow of 200 sccm of Ar, 100 sccm of H_2_, and 1.7 sccm of CH_4_ (99.999%, 99.999%, and 99.99% purity, respectively) is established in the tube at room temperature (Supplementary Fig. 1f). The furnace is on a sliding track. It is initially slid away from the substrates, and all zones are preheated to 920 °C. The nanoribbon growth stage is then commenced by sliding the furnace over the substrates (Supplementary Fig. 1g). As substrate temperature increases over the course of several minutes, nanoribbon evolution does not appreciably begin until the substrate temperature approaches 920 °C due to the strongly activated nature of graphene growth on Ge(001)^30–32^. To quantify when nanoribbon evolution effectively begins, we use the fact that nanoribbon length increases linearly with time. We plot nanoribbon length versus time elapsed since sliding the furnace over the substrates in Supplementary Fig. 21. We then fit these data to a line and extrapolate to find when the nanoribbons effectively begin growing, which we define as occurring when the length of the nanoribbons is equivalent to the characteristic seed size of 1.7 nm (i.e., when the length and width are both the size of the characteristic seed). The nanoribbons effectively begin growing 7 min after sliding the furnace over the substrate (Supplementary Fig. 20). Therefore, we define this instance 7 minutes after sliding the furnace as time = 0 for nanoribbon evolution. All CH_4_ exposure times specified throughout the manuscript are specified with respect to this instance (i.e., the specified CH_4_ exposure time is 7 min less than the time elapsed since sliding the furnace). The growth stage is terminated by sliding the furnace away from substrates to rapidly cool them in the same environment.

### Microscopy

After growth, the samples are characterized via SEM (Zeiss LEO 1530) to acquire basic length, width, and density information. STM is performed by transferring the nanoribbon samples through ambient air to a nitrogen glovebox, where they are sealed. The samples are later mounted to sample holders, loaded into an ultrahigh vacuum preparation chamber where they are annealed for 12 h at 400 °C, and imaged by STM (Omicron VT, base pressure of 1 × 10^−11^ mbar). The STM imaging is performed at room temperature using electrochemically etched W tips. Scanning tunneling spectroscopy is performed using a lock-in amplifier with a dither frequency of 10 kHz and modulation amplitude of 30 mV.

### Fabrication and characterization of graphene nanoribbon FETs

After synthesis, the nanoribbons are transferred to SiO_2_ on Si with a copolymer of crosslinkable poly(methyl methacrylate) (PMMA)^66^. The copolymer consisting of 96 mol% methyl methacrylate (MMA) with 4 mol% of thermally crosslinkable glycidyl methacrylate (GMA), PMMA-GMA (96% PMMA, 4% GMA), is spin-coated on the sample, and the films are thermally annealed at 160 °C for 3 h in a vacuum to promote better bonding of the copolymer with the nanoribbon/Ge substrate. Excess polymer is removed by rinsing in toluene, resulting in a film that is 3–5 nm in thickness. Additional PMMA is spin-coated on top of the PMMA-GMA film, and the substrate is annealed at 160 °C for 5 h in an N_2_ environment (<1 ppm O_2_ and <1 ppm H_2_O). The backside of the sample that is uncoated with polymer undergoes an O_2_ plasma etch (50 W, 10 mTorr, 10 sccm of O_2_) for 3 min (Unaxis 790 Reactive Ion Etcher) to remove graphene. The sample is then floated on 3:1:1 H_2_O:HF:H_2_O_2_ to etch the Ge substrate. The nanoribbon/polymer membrane is transferred from the Ge etchant to three successive H_2_O baths and finally to a piranha cleaned SiO_2_ (15 nm) on Si substrate. The substrate is spin dried and then annealed at 120 °C for 3 min in an N_2_ environment (<1 ppm O_2_ and <1 ppm H_2_O). The substrate is soaked in acetone at room temperature for ~1 h and is subsequently thermally annealed in a horizontal quartz tube furnace at 400 °C for 1 h at ~10^−6^ torr to remove polymer residue.

To fabricate graphene nanoribbon field-effect transistors, source and drain contacts are patterned via electron-beam lithography, development, and thermal evaporation of Cr/Pd/Au (thicknesses of 0.7/9.3/10 nm). The 15 nm of SiO_2_ is used as the gate dielectric, and the degenerately doped Si substrate serves as the back gate electrode. Devices are measured in ambient laboratory conditions at room temperature using a Keithley 2636A SourceMeter. The transistors exhibit hysteresis in the *I*_ds_ versus *V*_gs_ characteristics, as expected for nanoribbon devices measured on SiO_2_ substrates in ambient air^67,68^. This hysteresis is observed in the full *I*_ds_ versus *V*_gs_ sweeps in Supplementary Fig. 17. Measurements of *I*_ds_ versus *V*_ds_ are provided in Supplementary Fig. 18.

## Supplementary information

Supplementary Information

## Data Availability

The data that support the findings of this study are presented in the main text and the Supplementary Information and are available from the corresponding author upon request.
