# Supplementary material for: Graphene nanoribbons initiated from molecularly derived seeds
Source: Nat Commun. 2022 May 30;13:2992. doi: 10.1038/s41467-022-30563-6 (PMC9151757; doi:10.1038/s41467-022-30563-6)
Supplement: Supplementary file 1 — Supplementary Information [file 41467_2022_30563_MOESM1_ESM.pdf]

Supplementary Information

Graphene nanoribbons initiated from molecularly derived seeds

A. J. Way et al.

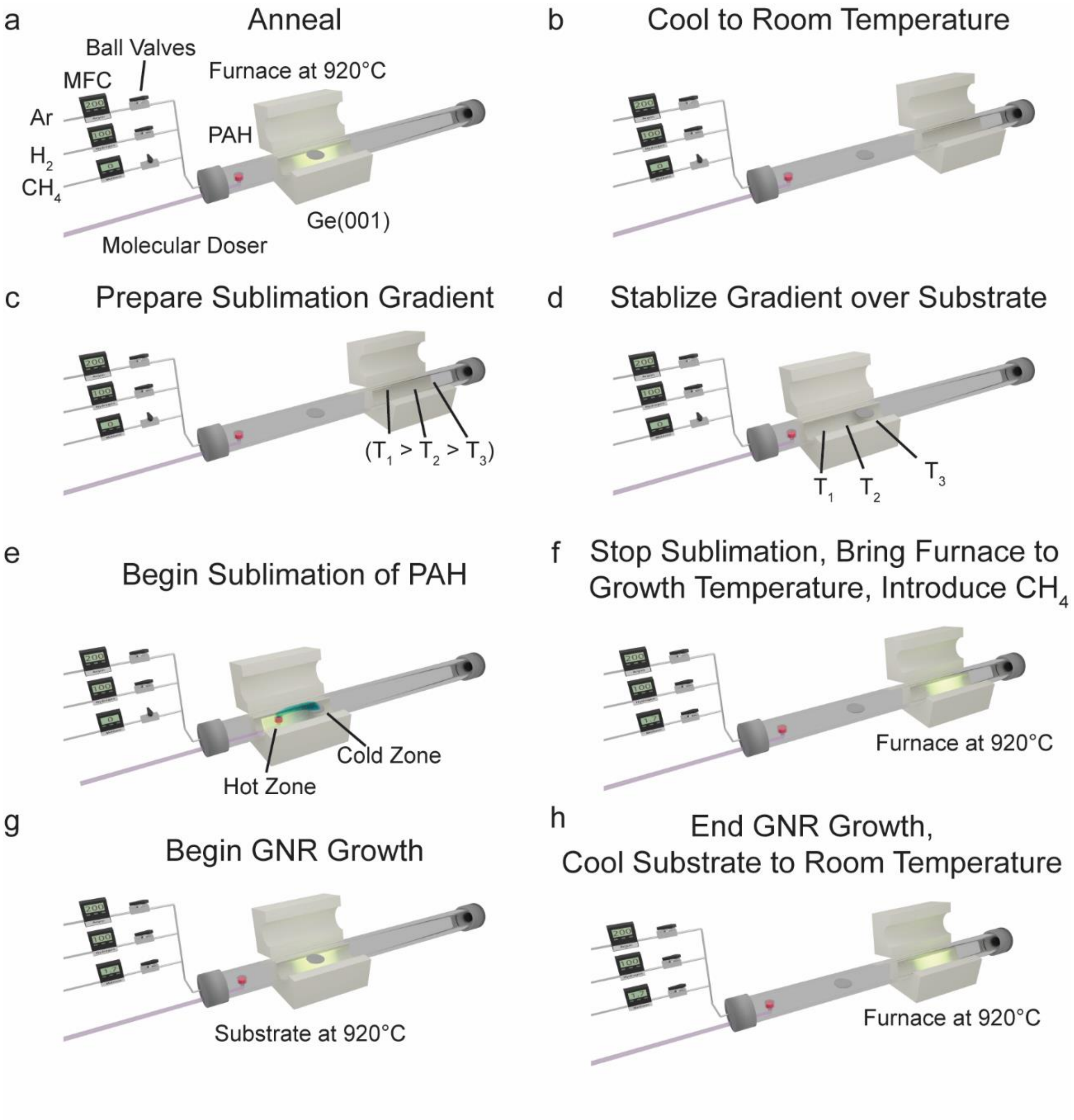

Supplementary Figure 1. Schematic of the growth procedure. a, The Ge(001) substrate is annealed in an atmosphere of 67% Ar and 33% H_2 at 920 °C for 1.5 h. **b**, The furnace is slid away from the substrate and the whole system is decreased to room temperature. **c**, A temperature gradient is created in the furnace. In the case of PTCDA, T_1 = 220 °C, T_2 = 180 °C, and T_3 = 165 °C. **d**, The furnace is slid back over the substrate and the gradient is stabilized. **e**, A quartz boat filled with PAH powder is pushed into the zone at T_1, which is the sublimation temperature of the PAH, while the substrate is at T_3, which is lower than the sublimation temperature and promotes deposition on the substrate. **f**, The furnace is slid

away from the boat and substrate to stop sublimation, and the substrate is cooled to room temperature. Additionally, CH_4 is introduced into the system, and the furnace temperature is increased to the growth temperature of 920 °C. **g**, The furnace is slid over the substrate to begin growth at 920 °C in an environment of 66% Ar, 33% H_2, and 0.56% CH_4. **h**, After the desired CH_4 exposure time (which is used to control the width and length of the nanoribbons), the furnace is slid away from the substrate to terminate growth.

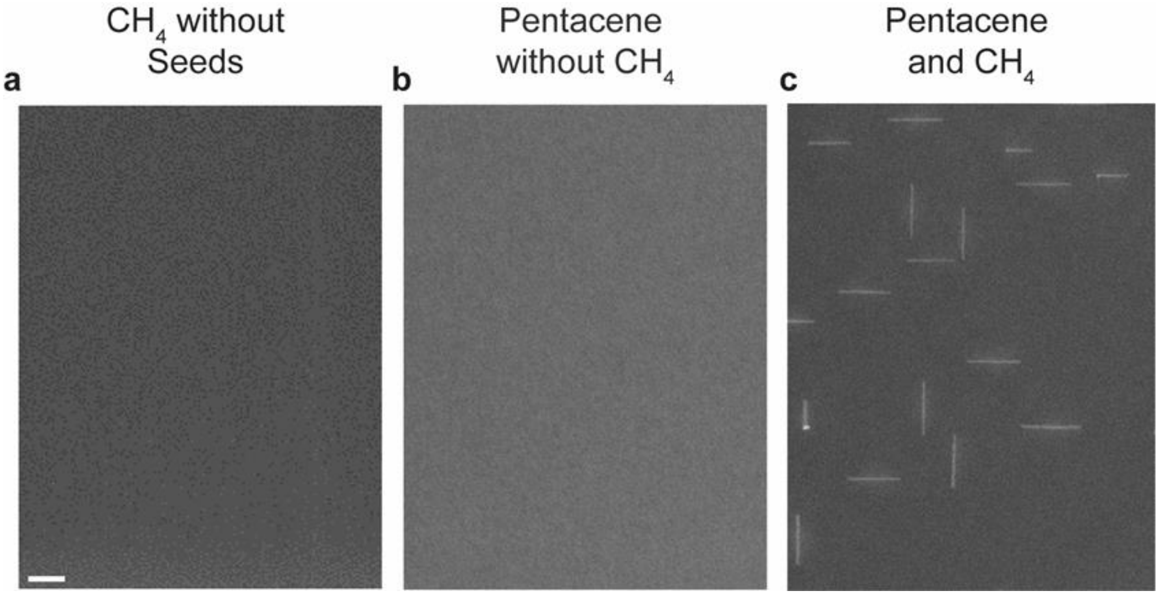

Supplementary Figure 2. Control experiments. a, Control experiment without the deposition of PAHs. No nanoribbons are observed, confirming that nanoribbons are unable to spontaneously nucleate from the supply of CH_4, alone, in the absence of PAH-derived seeds. **b**, Control experiment that forgoes the delivery of CH_4 to the Ge substrate after deposition of pentacene-derived seeds. No nanoribbons are observed, confirming that PAHs themselves are unable to form nanoribbons. **c**, Control experiment showing that nanoribbons are obtained only after both dosing the surface with pentacene molecules and evolving them from CH_4. *Annealing*: All samples annealed with the same protocol described in the body of the manuscript. *Dosing with PAH stage*: All samples are brought to the same temperature for PAH dosing. However, no pentacene powder is introduced for sample (**a**), whereas the same dose of pentacene is applied to both (**b**) and (**c**). *Anisotropic growth stage*: All samples are subjected to the same growth stage temperature of 920 ˚C and duration of 173 min. The CH_4 growth concentration is 0.56% in (**a**) and (**c**) but 0% in (**b**). Scale bar is 200 nm and applies to all three SEM images.

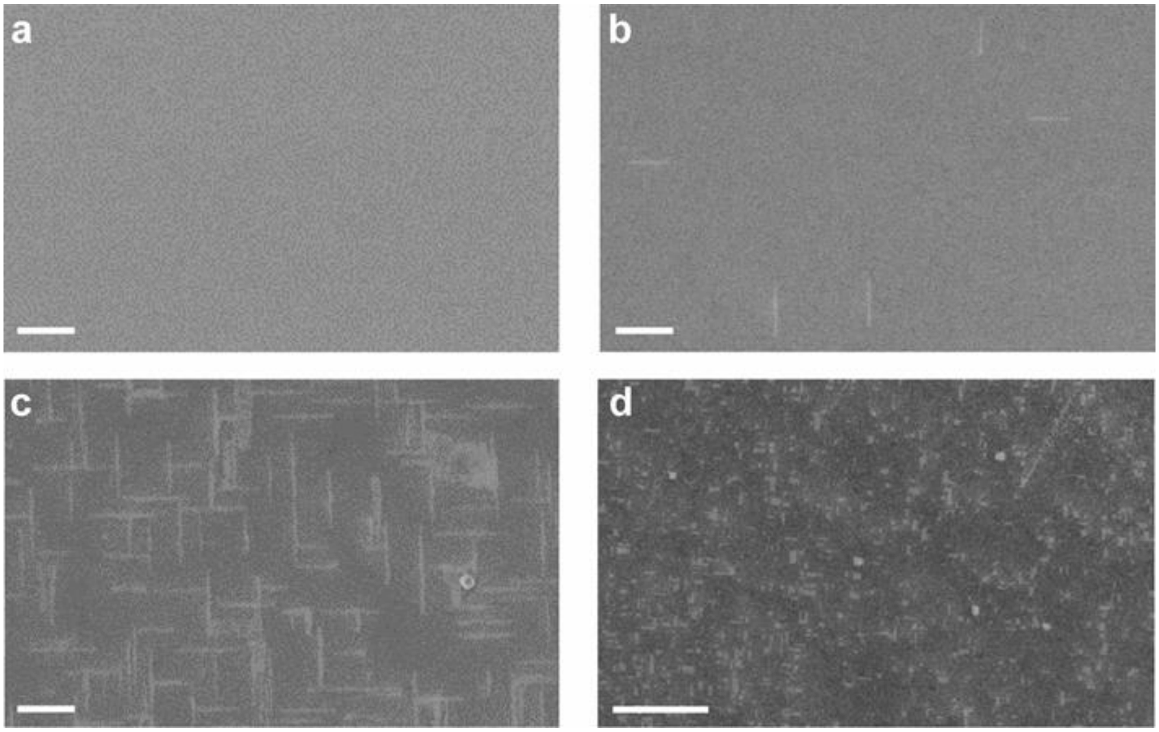

Supplementary Figure 3. **Tuning nanoribbon density by varying PAH dose. a-d**, SEM images of syntheses conducted under the same anisotropic growth condition with 173 min of CH_4 exposure, but with increasing dose of pentacene by increasing the sublimation temperature. Sublimation temperature = 141, 158, 165, and 182 °C in (**a**), (**b**), (**c**), and (**d**), respectively. Sublimation time is 10 min. Image (**a**) shows no nanoribbons, (**b**) shows low density of nanoribbons, (**c**) shows high density of nanoribbons, and (**d**) shows extremely high density of nanoribbons that are merging to form a disordered film of graphene with near continuous coverage. Scale bars are 200 nm (**a-c**) and 1 μm (**d**).

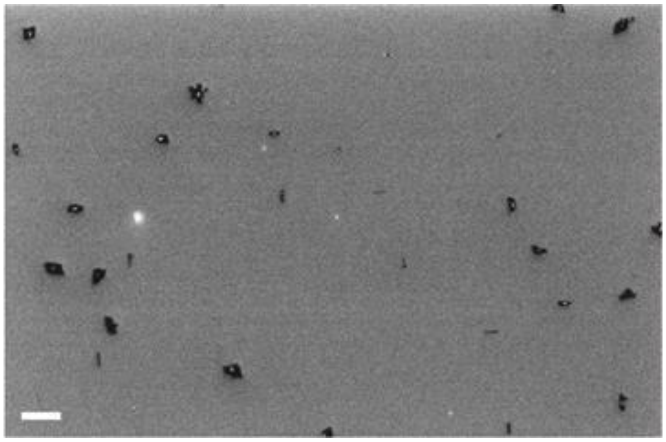

Supplementary Figure 4. Burst nucleation control. Evolution of low-aspect ratio crystals (observed as dark features) using burst of CH_4 in failed attempt to initiate nanoribbon nucleation. Scale bar is 1 μm. Instead of dosing with PAH, the substrate is exposed to a burst of 1.9% CH_4 for 10 min at 910 ˚C (above the critical concentration of CH_4 needed to spontaneously nucleate nanoribbons without seeds). The image above is taken immediately after this burst. Low-aspect ratio graphene crystals evolve rapidly during the burst, likely because of the superlinear dependence of growth rate on CH_4 concentration[1-3].

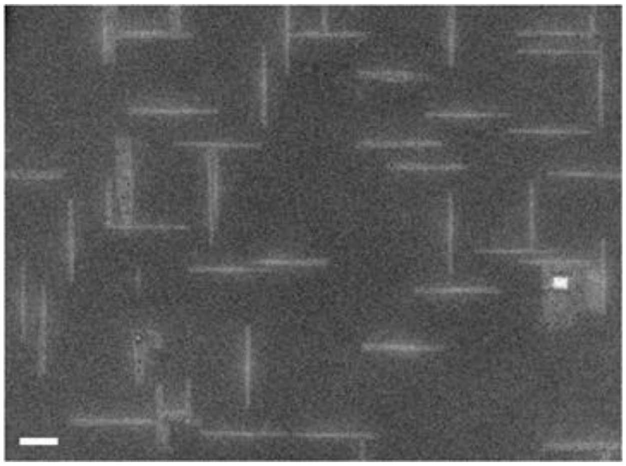

Supplementary Figure 5. Continuation of the series presented in Fig. 2a-e, showing graphene nanoribbons initiated from PTCDA-derived seeds after 203 min of exposure to CH_4. Scale bar is 200 nm.

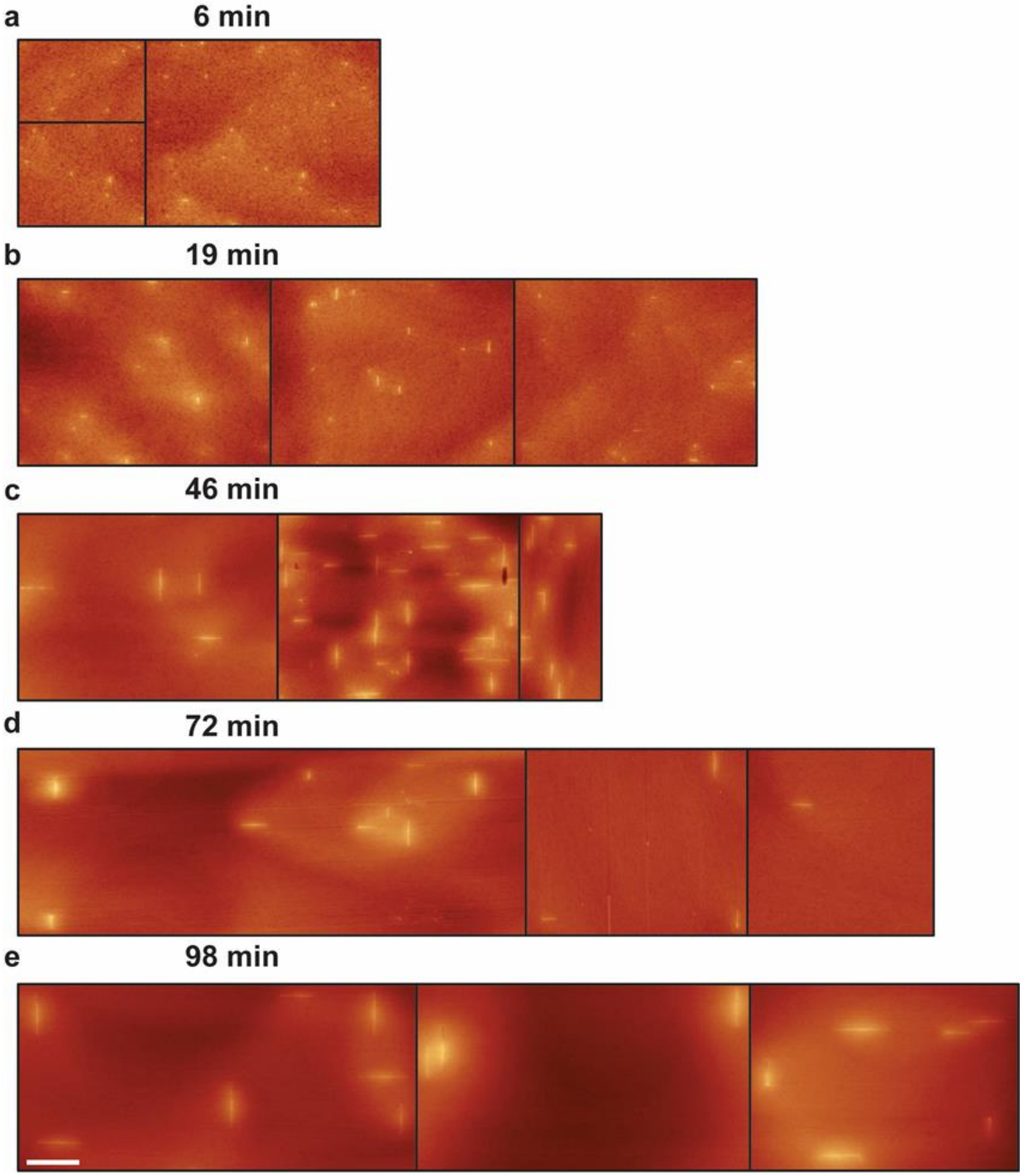

Supplementary Figure 6. Large-area STM images of PTCDA seeded graphene nanoribbons.

Compilation of large-area STM images for PTCDA seeded graphene nanoribbons grown for 6 (**a**), 19 (**b**), 46 (**c**), 72 (**d**), and 98 (**e**) min (applied bias = 2 V, tunneling current = 0.1 nA). Scale bar is 200 nm and applies to all images. The spatial variation of the background Ge topography arises from nanoribbon

induced surface roughening, as reported previously[2]. Color is scaled to topographic height, with dark red being lowest and light yellow being highest.

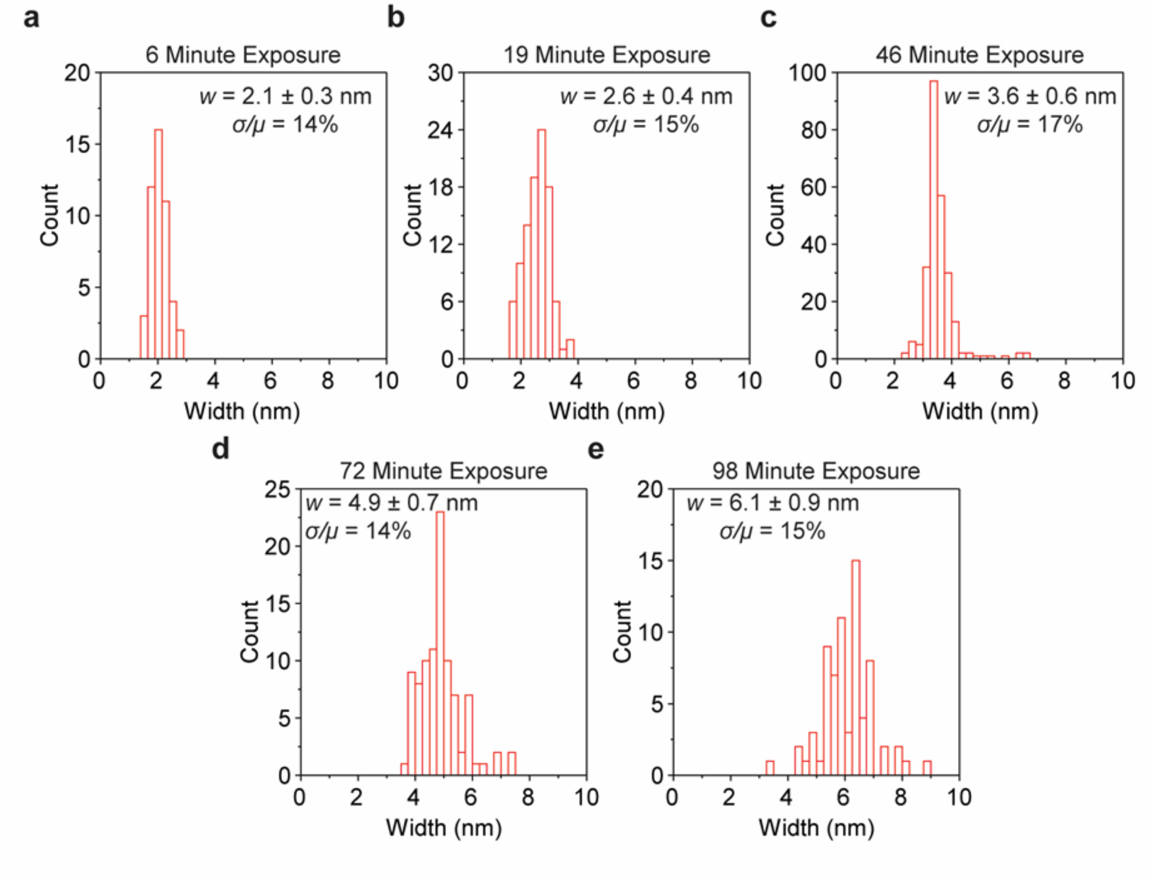

Supplementary Figure 7. Nanoribbon width distribution. Further characterization of nanoribbon samples analyzed in Fig. 2 (initiated from PTCDA-derived seeds). Nanoribbon width distributions after 6 (**a**), 19 (**b**), 46 (**c**), 72 (**d**), and 98 (**e**) min of evolution from CH_4. The mean, standard deviation, and polydispersity (σ/μ) are indicated in each panel.

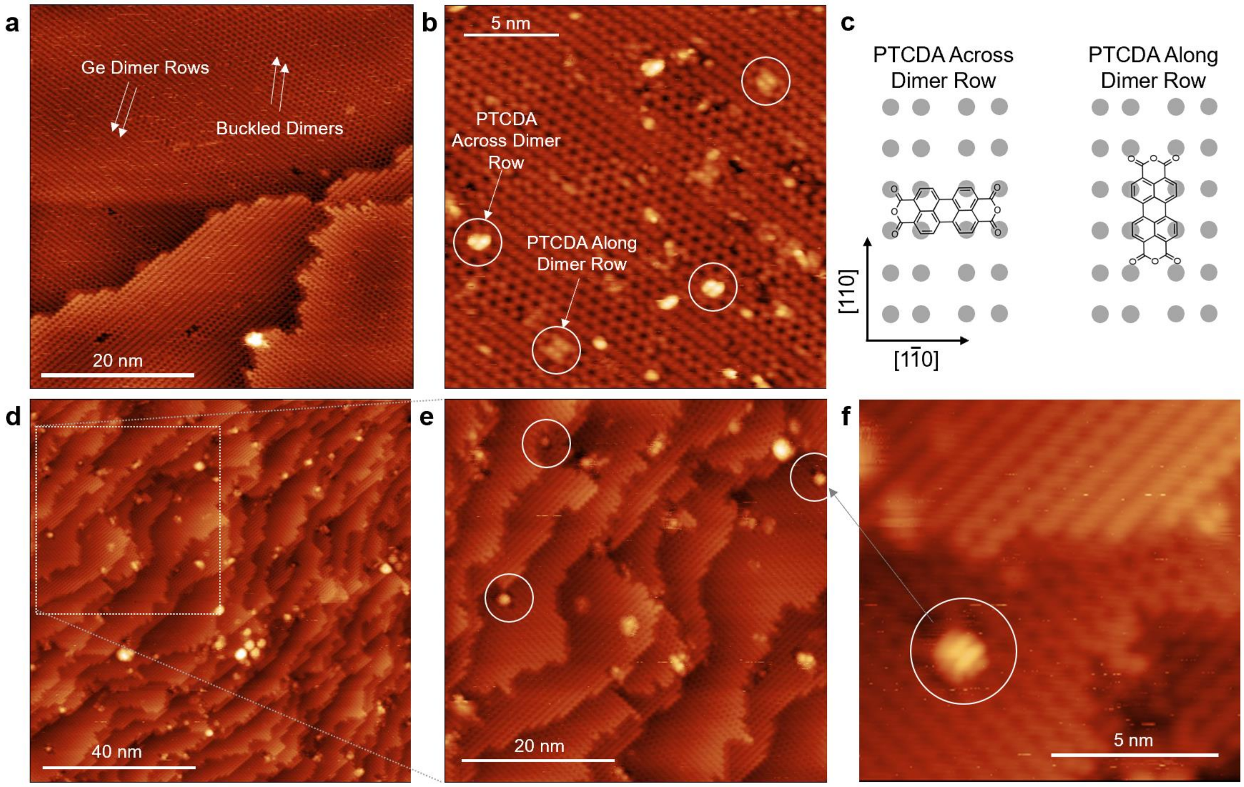

Supplementary Figure 8. STM of PTCDA deposited *in situ* within STM chamber before and after high temperature anneal (without CH_4 exposure). a, STM of the Ge(001) surface after sputter cleaning and annealing in ultrahigh vacuum (UHV) at ~900 ˚C for 1 hr. Ge dimer rows and buckled dimers are highlighted. **b,** STM of the Ge(001) surface after deposition of PTCDA molecules from an *in situ* thermal crucible dosing source. Primarily two orientations of PTCDA molecules are observed, circled in white. **c**, Schematic diagram of two predominant orientations on the Ge(001) surface. Orientation assignments and schematic diagram based on Supplementary Ref. 4. Atomic coordinates not precise; and schematic diagram not to scale. **d,** Large area STM image of the Ge surface dosed with PTCDA characterized in part b, after flash annealing at ~900 ˚C for ~10 minutes, evidencing the survival of carbon species (bright features). **e,** Zoomed in STM image of part d, evidencing various features on the surface possessing a diameter of ~1.5 – 2.5 nm, including some partially embedded in the Ge surface (circled). **f,** Higher resolution characterization of one of the partially embedded features. Color in **a, b, d, e, f** is scaled to topographic height, with dark red being lowest and light yellow being highest.

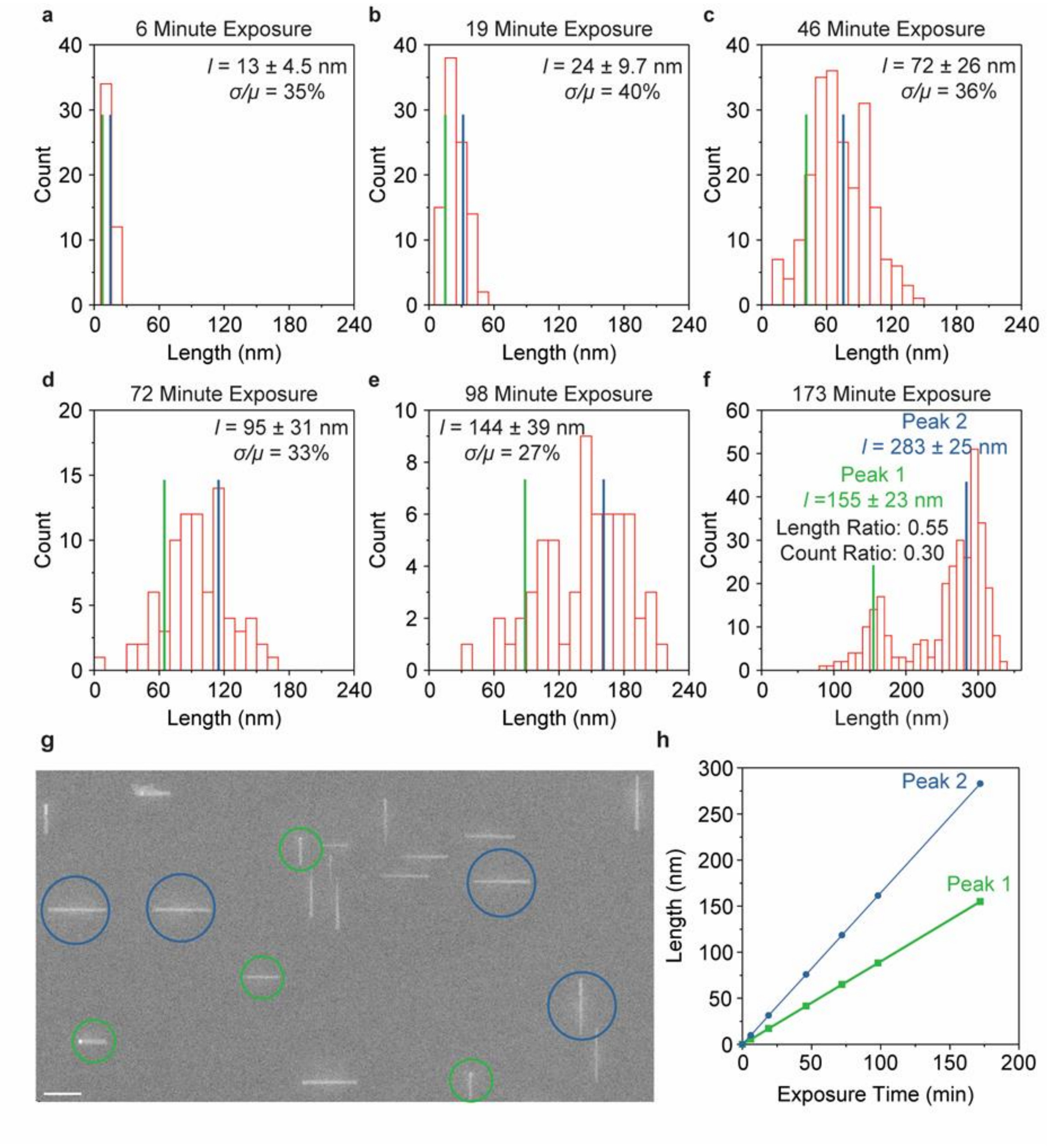

Supplementary Figure 9. Nanoribbon length distribution. Further characterization of nanoribbon samples analyzed in Fig. 2 (initiated from PTCDA-derived seeds). **a-e**, Nanoribbon length distributions after 6 (**a**), 19 (**b**), 46 (**c**), 72 (**d**), and 98 (**e**) min of evolution from CH_4. **f**, Nanoribbon length distribution of an additional sample after 173 min of evolution from CH_4. The mean, standard deviation, and polydispersity (σ/μ) are indicated in each panel. The bimodal nature of the nanoribbon length distribution is clearly observed in (**f**), in which the two modes are marked as Peak 1 (green) and Peak 2 (blue). A

mean length ratio of 0.55 is observed when comparing the two distributions. **g**, SEM image of the 173 min growth sample with Peak 1 nanoribbons circled in green and Peak 2 nanoribbons circled in blue. Scale bar is 200 nm. **h**, Expected length versus exposure time for Peak 1 (green line) and Peak 2 (blue line) nanoribbons after linear extrapolation between the origin and the mean length of each distribution from the 173 min exposure. The expected mean length of each distribution derived from this extrapolation is denoted by vertical green and blue lines in parts (**a**) through (**e**).

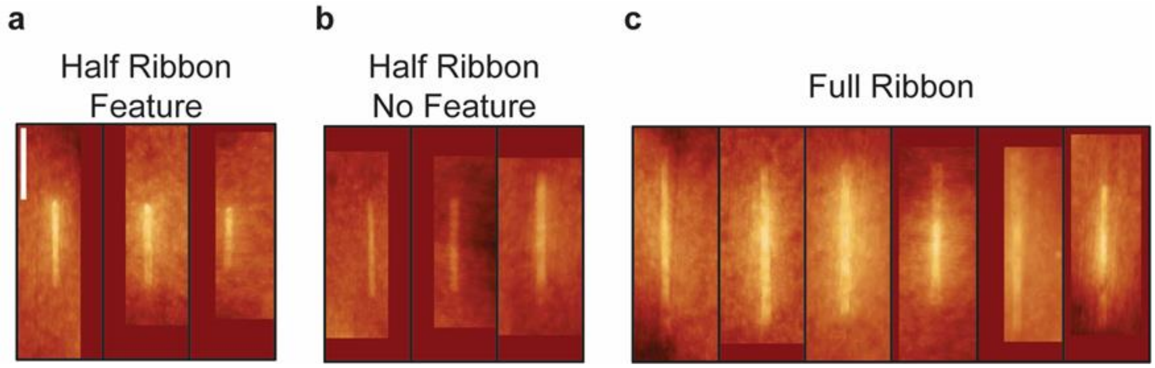

Supplementary Figure 10. STM of features related to the bimodal length distribution. There is no clear difference between half- versus full-length nanoribbons. In some instances, half-length ribbons appear to have an elevated feature at one end of the ribbon (top side of ribbon) as shown in (**a**), but in other instances this elevated feature is not apparent (**b**). For comparison, an elevated feature appears in the middle of each full-length ribbon (**c**). All nanoribbons initiated from PTCDA-derived seeds and evolved via 46 min of CH_4 exposure. Scale bar is 50 nm and applies to all images. Color is scaled to topographic height, with dark red being lowest and light yellow being highest.

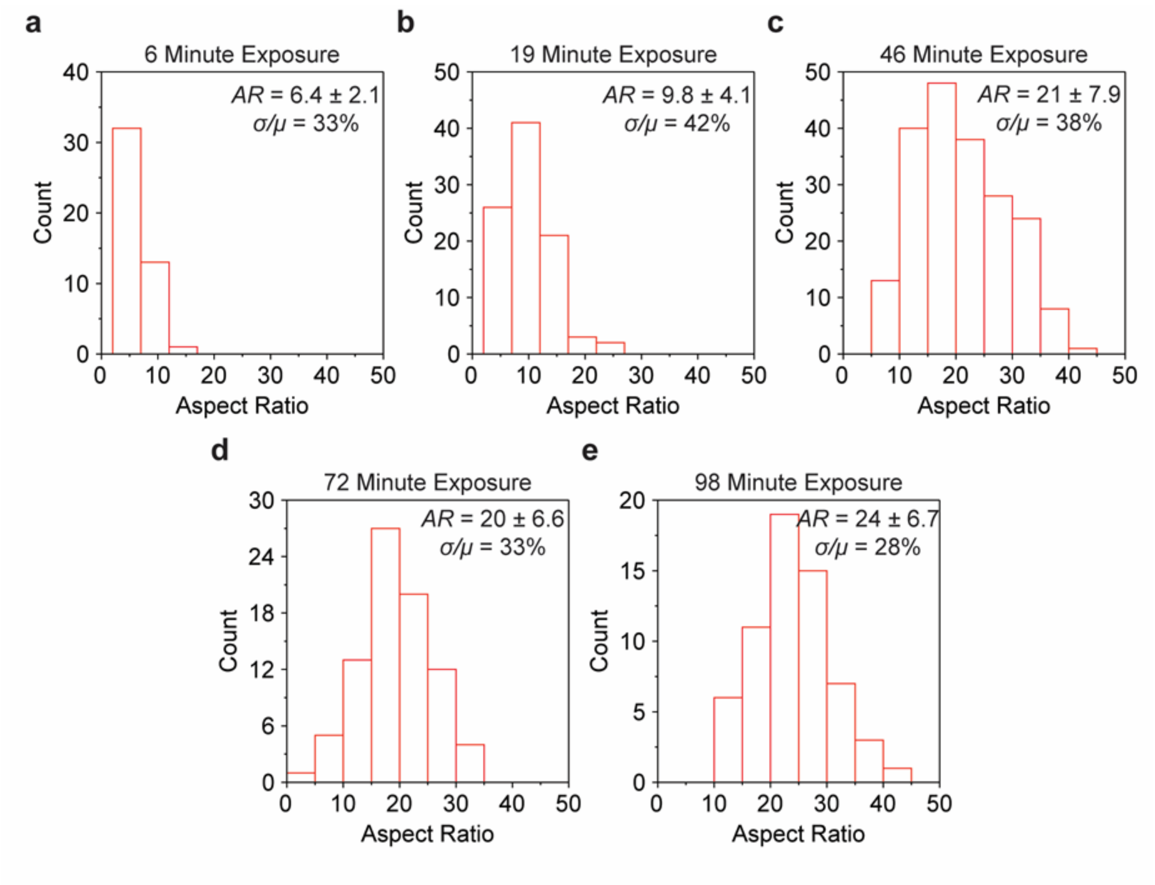

Supplementary Figure 11. Aspect ratio distribution. Further characterization of nanoribbon samples analyzed in Fig. 2 (initiated from PTCDA-derived seeds). **a-e**, Nanoribbon aspect ratio (*AR*) after 6 (**a**), 19 (**b**), 46 (**c**), 72 (**d**), and 98 (**e**) min of evolution from CH_4. The mean, standard deviation, and polydispersity (σ/μ) are indicated in each panel.

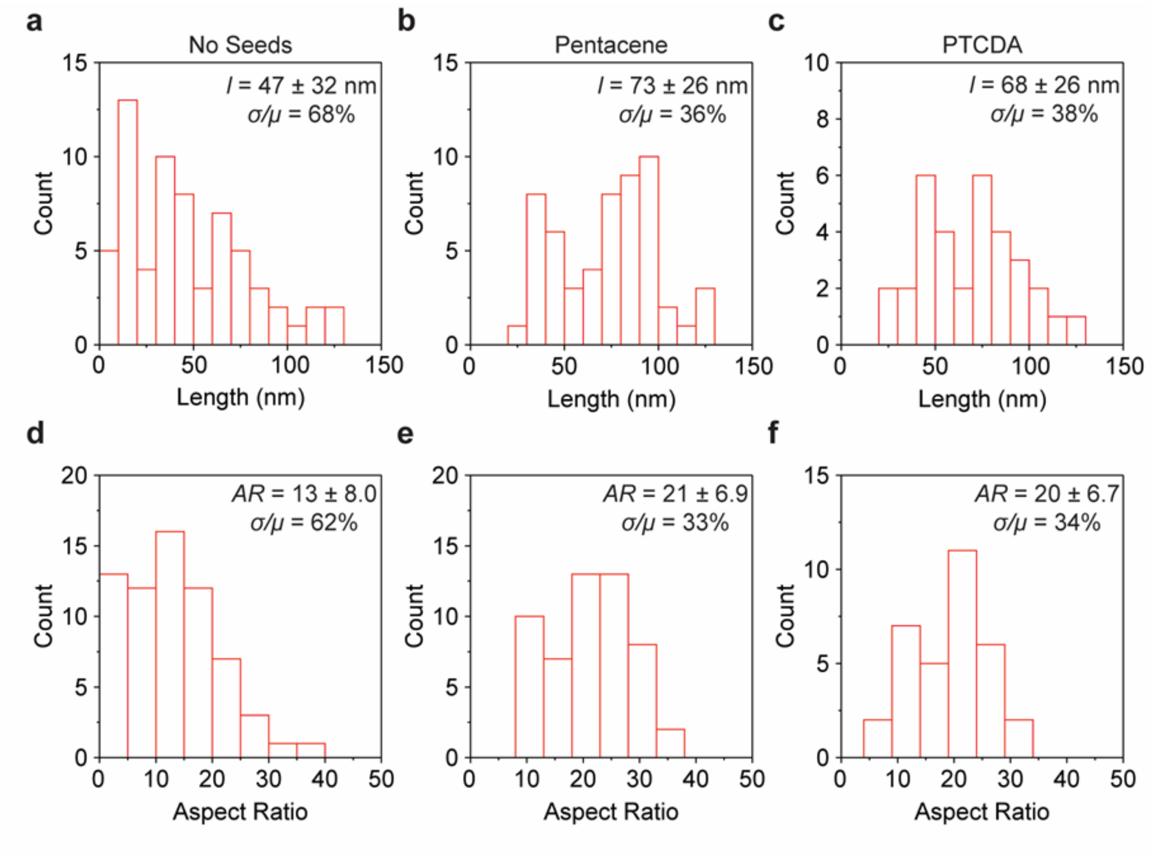

Supplementary Figure 12. Additional length and aspect ratio distributions. Further characterization of nanoribbon samples analyzed in Fig. 4 (initiated from PTCDA-derived seeds). Length and aspect ratio histograms for nanoribbons initiated without seeds (**a**,**d**), via pentacene-derived seeds (**b**,**e**), and via PTCDA-derived seeds (**c**,**f**). The mean, standard deviation, and polydispersity (σ/μ) are indicated in each panel.

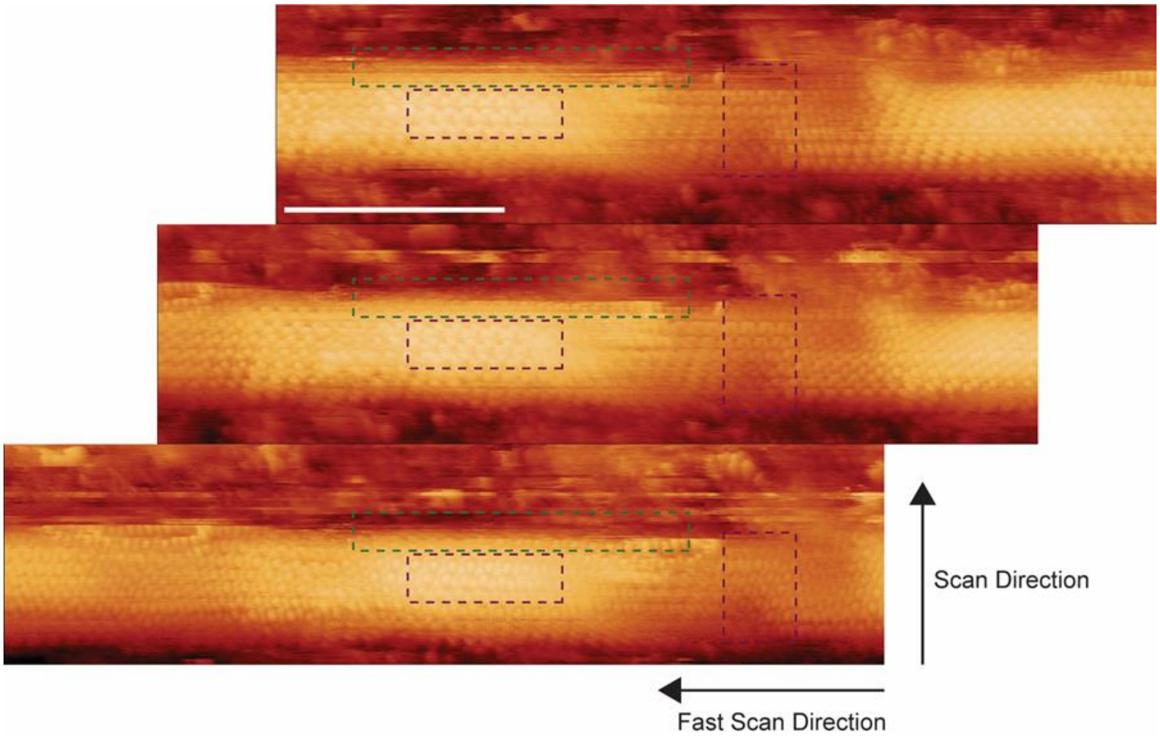

Supplementary Figure 13. **Multiple sequential high-resolution STM images.** Scans of the same nanoribbon characterized in Fig. 4c (applied bias = 0.2 V, tunneling current = 1.0 nA). Scale bar is 5 nm. These images demonstrate that the quasiparticle interference patterns in the nanoribbon interiors (purple dashed boxes) and the apparent structure of the edges (green dashed boxes) can change from scan to scan. These changes likely arise from extrinsic factors that affect the image (but not the nanoribbon), including changes in GeO_x species and adsorbates near the edges and/or adsorption/desorption from the tip. Color is scaled to topographic height, with dark red being lowest and light yellow being highest.

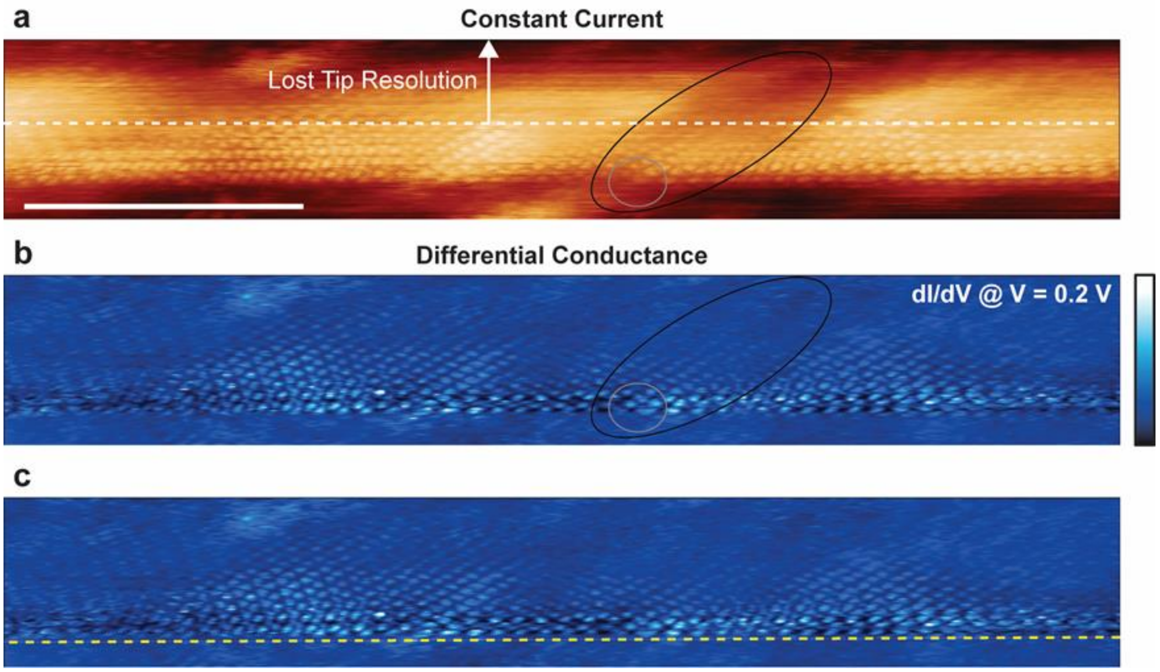

Supplementary Figure 14. Additional high-resolution STM scans. High-resolution STM constant current (**a**) and differential conductance (**b**,**c**) images of another nanoribbon initiated from pentacene-derived seeds after 46 min of evolution from CH_4 (applied bias = 0.2 V, tunneling current = 1.0 nA). Scale bar is 5 nm. The color in **a** is scaled to topographic height, with dark red being lowest and light yellow being highest. The color in **b,c** is scaled to differential conductance, with lighter color being higher conductance and darker color being lower conductance. High-resolution is obtained only in the bottom-half of each image. The image in part **c** is a replica of the image in part **b**, with the addition of a horizontal line to guide the eye. The data qualitatively show that the line edge roughness is low and demonstrate faceted edge segments greater than 5 nm in length. A feature that is a possible edge kink is circled in grey in (**a**) and (**b**). The location of this possible kink also coincides with a depression in the substrate topography (circled in black), possibly producing an imaging artifact that appears as an edge kink or possibly evidencing a link between substrate topography and edge kink formation or stabilization.

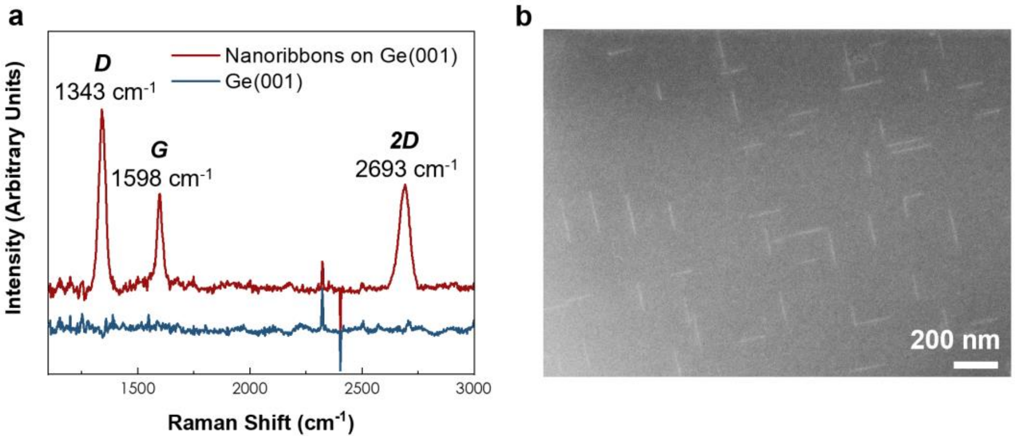

Supplementary Figure 15. Raman spectrum of graphene nanoribbons (initiated from PTCDA-derived seeds and grown with 98 minutes of CH_4 exposure time) on Ge(001) growth substrate. (**a**) D- and G-band scattering are observed in the nanoribbon sample at roughly 1343 and 1598 cm^{-1}. 2D-scattering is observed at roughly 2693 cm^{-1}. All Raman data acquired with 532 nm excition wavelength using a Horiba LabRAM HR Evolution Raman Microscope. (**b**) Scanning electron micrograph of area characterized in part (a).

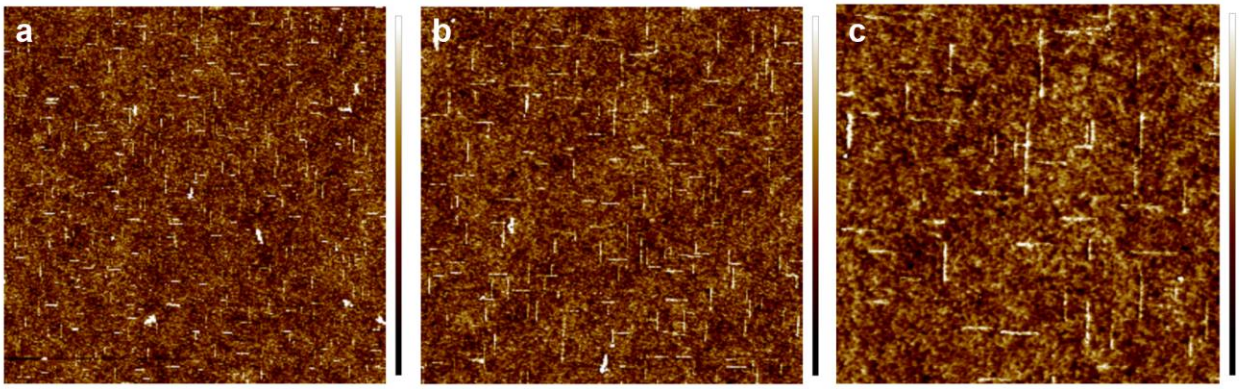

Supplementary Figure 16. Atomic force micrographs of graphene nanoribbons (initiated from PTCDA-derived seeds and grown with 98 minutes of CH_4 exposure time) transferred onto 15 nm SiO_2 on Si substrates. Characterization of 5 × 5, 3 × 3, and 1.5 × 1.5 μm^2 areas are shown in **(a)**, **(b)**, and **(c)**, respectively. Ranges of height scale-bar (denoted by color) are 1.7, 1.8, and 1.8 nm, respectively. Data acquired using Bruker Icon AFM (tapping mode).

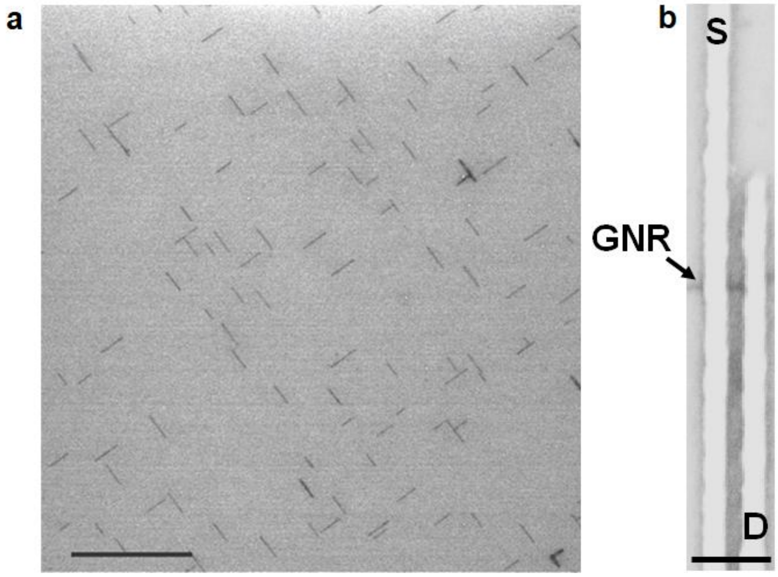

Supplementary Figure 17. Images of transferred nanoribbons and FET. a, SEM image of nanoribbons transferred to SiO_2 with the approach reported in the Methods section. Scale bar is 1 µm. **b,** Representative SEM image of nanoribbon FET with graphene nanoribbon (GNR) and source (S) and drain (D) electrodes labeled. Substrate is 15 nm SiO_2 (back-gate dielectric) on highly doped Si (back-gate electrode). Nanoribbon width in this FET is ~10 nm wide to aid in visualization. Scale bar is 100 nm.

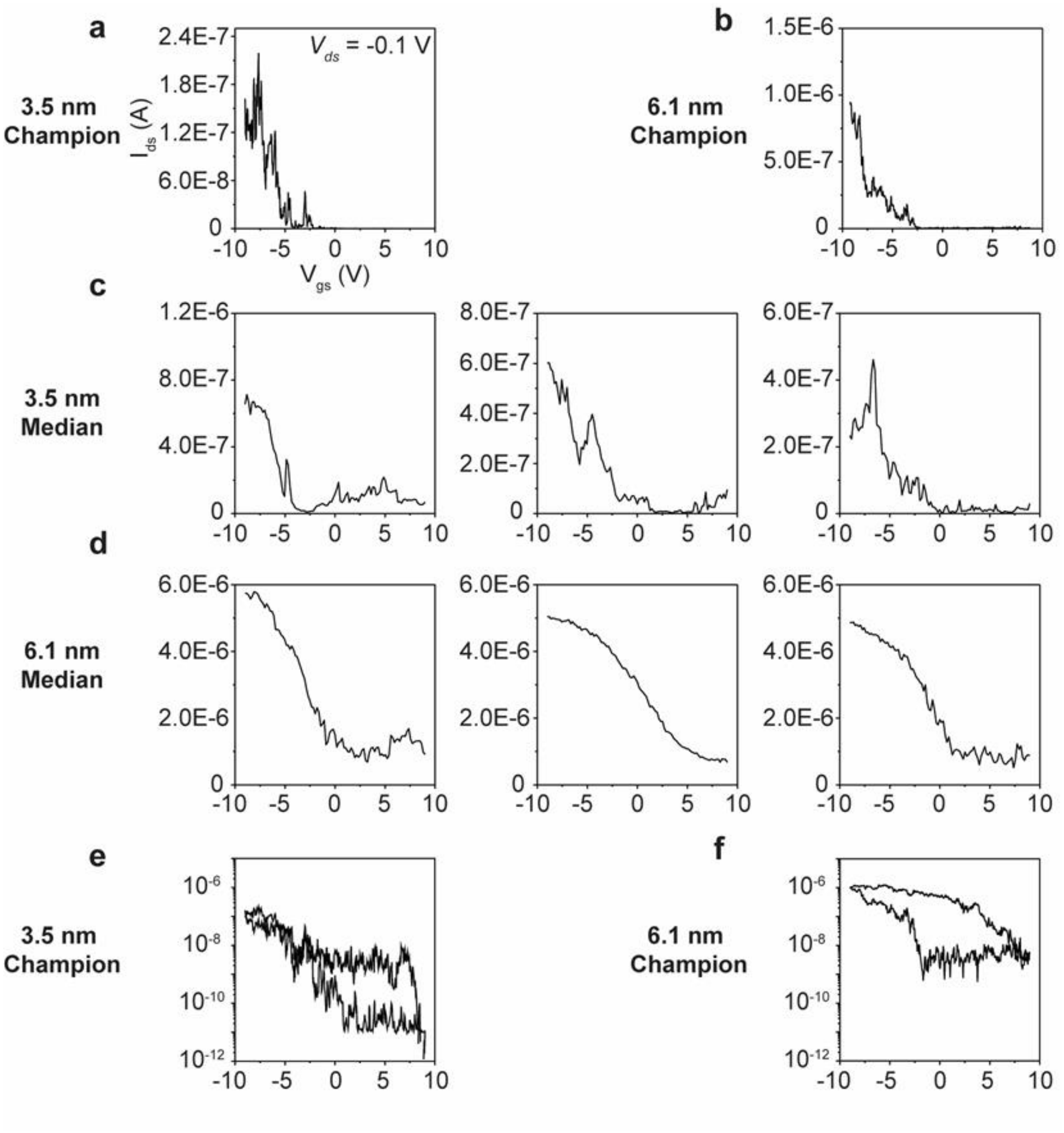

Supplementary Figure 18. Linear and forward/reverse sweeps of FET data. Additional analysis of data presented in Figure 5 on linear scales (**a-d**) and comparing forward and reserve sweeps (**e-f**). The plots show I_{ds} versus V_{gs} for the forward sweep of the champion (**a**,**b**) and median (**c**,**d**) nanoribbon FETs possessing widths of either 6.1 (**b**,**d**) or 3.5 nm (**a**,**c**). Measurements are made with V_{ds} = -0.1 V on 15 nm of SiO_2 on Si. All FETs are measured at room temperature in ambient laboratory conditions. **e**,**f**, Plot of I_{ds} versus V_{gs} for the forward (V_{gs} is swept negative to positive) and reverse (V_{gs} is swept positive to negative)

sweeps of the champion nanoribbon FETs. The FETs exhibit hysteresis as expected for nanoribbons FETs on SiO_2 measured in ambient air.[5,6]

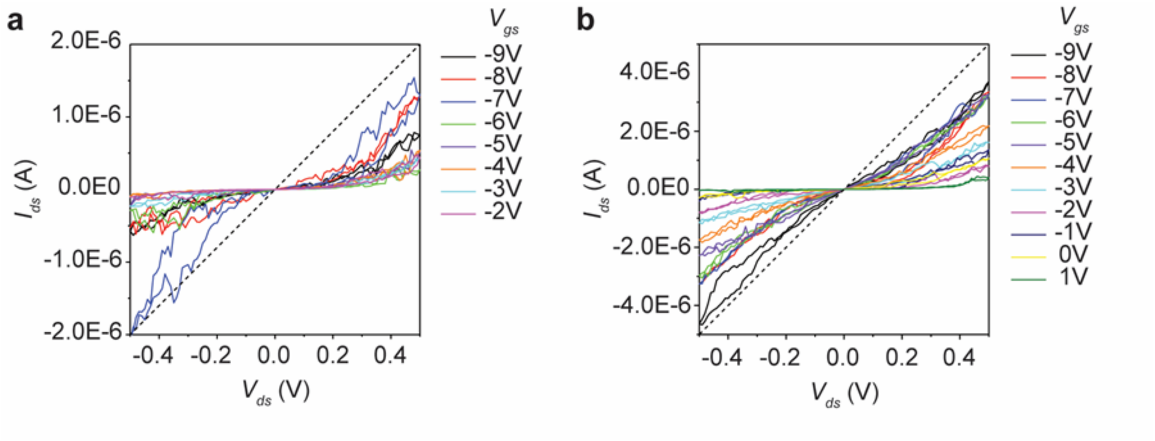

Supplementary Figure 19. Electrical performance of champion devices. I_{ds} versus V_{ds} curves of champion FETs shown in Figure 5 for nanoribbons of width 3.5 nm (**a**) and 6.1 nm (**b**).

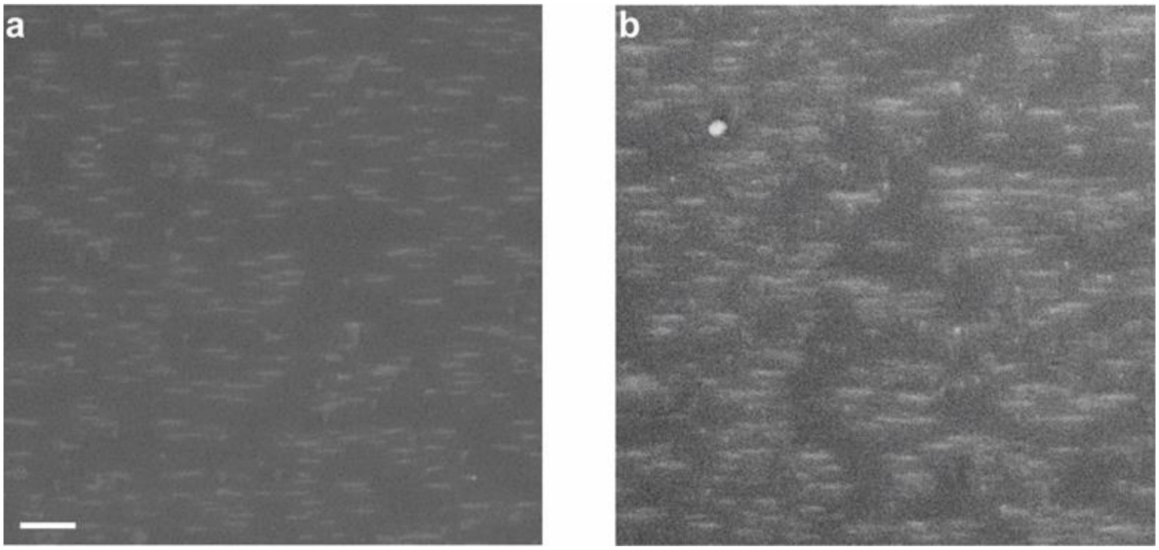

Supplementary Figure 20. Molecular seeding on vicinal Ge. SEM images of nanoribbons grown from PTCDA-derived seeds on vicinal Ge(001) surfaces (with 9˚ miscut towards the Ge[110] direction) after 23 (**a**) and 53 (**b**) min of evolution from CH_4. Scale bar in (**a**) is 200 nm and applies to both images.

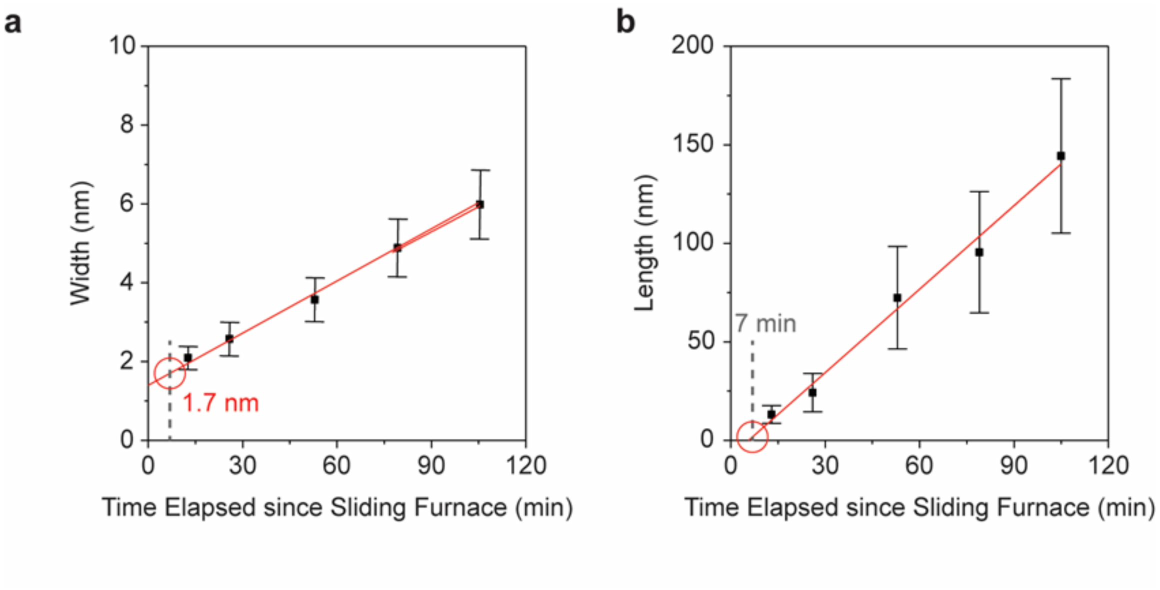

Suplementary Figure 21. Determining exposure time before growth begins. Width (**a**) and length (**b**) versus time elapsed since sliding furnace, showing that nanoribbon evolution does not begin immediately due to the time needed to reach the growth temperature of 920 ˚C. The length is 1.7 nm in part **b** at a time of 7 min, which quantifies the time needed to reach the growth temperature. The CH_4 exposure time presented in the main text is the time elapsed since sliding the furnace minus 7 min. Error bars are standard deviation of width (**a**) and length (**b**).

Supplementary References

(1) Jacobberger, R. M. et al. Direct oriented growth of armchair graphene nanoribbons on germanium. *Nat. Commun.* **6**, 8006 (2015).

(2) Saraswat, V. et al. Synthesis of armchair graphene nanoribbons on germanium-on-silicon. *J. Phys. Chem. C* **123**, 18445–18454 (2019).

(3) Jacobberger, R. M. et al. Alignment of semiconducting graphene nanoribbons on vicinal Ge(001). *Nanoscale* **11**, 4864−4875 (2019).

(4) Kocan, P. et al. Adsoprtion of PTCDA on Ge(001). *J. Phys. Chem. C* **121**, 3320-3326 (2017).

(5) Lee, J. S. et al. Origin of gate hysteresis in carbon nanotube field-effect transistors. *J. Phys. Chem. C* **111**, 12504−12507 (2007).

(6) Kim, W. et al. Hysteresis caused by water molecules in carbon nanotube field-effect transistors. *Nano Lett.* **3**, 193−198 (2003).
